# Extracting Conditionally Heteroskedastic Components using Independent Component Analysis

**DOI:** 10.1111/jtsa.12505

**Published:** 2019-09-08

**Authors:** Jari Miettinen, Markus Matilainen, Klaus Nordhausen, Sara Taskinen

**Affiliations:** ^1^ Department of Signal Processing and Acoustics Aalto University Helsinki Finland; ^2^ Department of Mathematics and Statistics University of Turku Turku Finland; ^3^ Institute of Statistics & Mathematical Methods in Economics Vienna University of Technology Wien Austria; ^4^ Department of Mathematics and Statistics University of Jyvaskyla Jyväskylä Finland; ^5^ Turku PET Centre Turku University Hospital and University of Turku Finland

**Keywords:** ARMA‐GARCH process, asymptotic normality, autocorrelation, blind source separation, principal volatility component

## Abstract

In the independent component model, the multivariate data are assumed to be a mixture of mutually independent latent components. The independent component analysis (ICA) then aims at estimating these latent components. In this article, we study an ICA method which combines the use of linear and quadratic autocorrelations to enable efficient estimation of various kinds of stationary time series. Statistical properties of the estimator are studied by finding its limiting distribution under general conditions, and the asymptotic variances are derived in the case of ARMA‐GARCH model. We use the asymptotic results and a finite sample simulation study to compare different choices of a weight coefficient. As it is often of interest to identify all those components which exhibit stochastic volatility features we suggest a test statistic for this problem. We also show that a slightly modified version of the principal volatility component analysis can be seen as an ICA method. Finally, we apply the estimators in analysing a data set which consists of time series of exchange rates of seven currencies to US dollar. Supporting information including proofs of the theorems is available online.

## 1 Introduction

The interest in modelling volatilities of financial time series has increased considerably since the introduction of autoregressive conditional heteroskedasticity (ARCH) (Engle, 1982) and generalized autoregressive conditional heteroskedasticity (GARCH) (Bollerslev, 1986) models. When several financial time series are analysed simultaneously and in particular, when the interest is in studying volatilities and co‐volatilities, multivariate GARCH (MGARCH) models offer popular tools for modelling. Two widely used MGARCH models are the VEC model (Bollerslev et al. 1988) and the BEKK model (Engle and Kroner, 1995), which differ in the parametrization of the conditional covariance matrix. A drawback of the MGARCH models is that the number of parameters increases rapidly with the number of time series (Bollerslev et al. 1994) and the estimation becomes infeasible. According to Chib et al. (2006), the models are thus often unreasonably simplified to enable efficient estimation.

Due to the ‘curse of dimensionality’ several dimension reduction techniques have been introduced in the literature. Among the most popular model‐based approaches is the *dynamic factor model* (DFM) considered in Forni et al. (2000), Bai and Ng (2002) and Forni et al. (2004) among others, which assumes that each time series in the data can be decomposed into two unobserved components: the common component driven by a very small number of common factors and the idiosyncratic component. The DFM is often defined using either static or lagged factor loadings, and depending on the assumptions made, the model can be fitted using principal component analysis (PCA) techniques in either time or frequency domain (Forni et al. 2000; Bai and Ng, 2002; Stock and Watson, 2002). For the recent extensions and developments of DFMs, see Fan et al. (2013), Barigozzi and Hallin (2016), Barigozzi and Hallin (2017) and references therein.

Another approach based on PCA was suggested in Hu and Tsay (2014), where a method called *principal volatility component* (PVC) analysis was introduced. PVC uses the so‐called generalized kurtosis matrix to detect linear combinations of multiple time series which do not have conditional heteroskedasticity, or equivalently, to identify common volatility factors. As shown later in this article, PVC is closely related to another widely used dimention reduction method for the analysis of multivariate financial data, that is, the *independent component analysis* (ICA). In ICA, the goal is to find a linear transformation of the multivariate data set which has mutually independent components (Hyvärinen and Oja, 2001; Nordhausen and Oja, 2018). The purpose of carrying out ICA can be to separate interesting components from the uninteresting ones, or to shift from a multivariate analysis to multiple univariate analyses. The latter is of particular interest when analysing multivariate time series. For approaches applying ICA in the context of MGARCH modelling, see Wu and Yu (2005), Matteson and Tsay (2011), García‐Ferrer et al. (2012) and Hai (2017), for example.

Most of the ICA methods rely on making the marginal densities of the components maximally non‐Gaussian. However, in the case of times series data, it is natural to make use of the temporal dependence of the components. Arguably, the most famous one of such methods is the second‐order blind identification (SOBI, Belouchrani et al., 1997) method, which performs approximate joint diagonalization of a set of autocovariance matrices for finding the transformation of the observed components into the independent components. SOBI performs well when the independent components have non‐zero linear autocorrelations, but it fails to utilize volatility clustering information. On the other hand, ICA methods which are tailored for separating time series with volatility clustering, see for example Hyvärinen (2001), Shi et al. (2009), Matilainen et al. (2015), Matilainen et al. (2017), do not utilize information coming from linear autocorrelations to their full extent.

In this article, we introduce the so‐called generalized SOBI (gSOBI) method, which uses both linear and quadratic autocorrelations to separate time series with or without volatility clustering from each other. We study the statistical properties of the gSOBI estimator and find its limiting distribution under general conditions. The asymptotic variances are derived in the case of an ARMA‐GARCH model. To the best of our knowledge, this is the first article to propose an ICA method which combines the use of linear and quadratic autocorrelations to separate various kinds of stationary time series. As another major contribution we provide tests for identifying latent components, which contain volatility clustering, and a criteria for ordering of components according to the ‘degree’ of volatility clustering. The development of such methods was motivated by Hu and Tsay (2014) who argued that especially in the case of multivariate econometric times series data, identifying and ordering such latent components is of main interest. Finally, we also show that PVC can be used to solve the ICA problem and suggest a small modification to make the method affine equivariant. Simulation studies and a real data example are used to compare the performance of the PVC estimator to the gSOBI estimator.

The article is organized as follows. In Section 2 we shortly define the independent component model and discuss some classical ICA methods for time series data. In Section 3 we recall the ARMA‐GARCH model which covers a wide collection of stationary time series. Section 4 includes the formal definition of the generalized SOBI estimator, asymptotic results of the estimator, and tests for linear autocorrelation and volatility clustering. In Section 5 we show that PVC can also be categorized as an ICA method utilizing volatility clustering. Finite sample properties of the gSOBI estimator, the PVC estimator and the tests are studied in Section 6. In Section 7, the methods are applied to a data set of exchange rates of seven currencies to US dollar, and the article is concluded in Section 8.

## 2 Independent Component Analysis

Let us first recall the independent component model, which states that the observable *p*‐variate time series ***x***: = (***x***_*t*_)_*t* = 0, ± 1, ± 2,…_ satisfy

$$x_{t}=\Omega s_{t},\;\;\;t=0,\pm 1,\pm 2,\text{…}$$

where **Ω** is a full‐rank *p* × *p* mixing matrix and ***s***: = (***s***_*t*_)_*t* = 0, ± 1, ± 2,…_ is a *p*‐variate latent time series. Given a realization ***x***_1_,…,***x***_*n*_ of the process (***x***_*t*_)_*t* = 0, ± 1, ± 2,…_, the aim of the ICA is to estimate the unmixing matrix **Γ**=**Ω** ^−1^ which transforms ***x*** back to the independent components ***s*** by ***s***=**Γ*x***. Under certain assumptions on ***s***, the unmixing matrix **Γ** is identifiable only up to scales, signs and order of its rows. The key assumptions are that the components of ***s*** are mutually independent and at most one of the components is i.i.d. Gaussian. Due to the scale ambiguity, we assume for simplicity that the components of ***s*** have unit variances.

Most of the ICA methods presented in the literature proceed in two steps, which are standardization (whitening) and rotation. Write now a standardized time series as

$$x_{t}^{st}=S^{-1/2}(x_{t})(x_{t}-E(x_{t})),$$

where ***S***(***x***_*t*_) denotes the covariance matrix functional of ***x***_*t*_. We then have that

$$s_{t}=Ux_{t}^{st}$$

for some orthogonal *p* × *p* matrix ***U*** (Cardoso and Souloumiac, 1993; Miettinen et al. 2015). The standardization thus solves the ICA problem up to rotation, that is, the final unmixing matrix is obtained as **Γ**=***US***(***x***_*t*_)^−1/2^.

The ICA literature comprises a vast amount of estimators with different strategies for finding the independent components. For an extensive overview of different estimation methods, see Comon and Jutten (2010). For the time being, the methods which use marginal densities of the components and ignore the temporal or spatial order of the components, have been the most popular choices for ICA applications. However, most often the ICA applications involve either time series or otherwise structured data, and it is evident that in such cases one can achieve better performance with methods utilizing these important data properties.

The standard precedure when investigating the temporal dependence in a univariate time series (*x*_*t*_)_*t* = 0, ± 1, ± 2,…_ is to compute autocovariances

$$E\left(\left(x_{t}-E(x_{t}))(x_{t+\tau }-E(x_{t+\tau }\right)\right),\;\tau >0.$$

If the autocovariances of the independent components are non‐zero, we can use joint diagonalization of autocovariance matrices to find the rotation matrix ***U*** as described in Tong et al. (1990) and Belouchrani et al. (1997). Notice, however, that zero autocovariances do not imply the absence of temporal dependence, as, for example, economic and financial time series often exhibit volatility clustering, that is, periods of lower and higher volatility. While the linear autocovariances might all be zero, the quadratic autocovariances

$$E\left({\left(x_{t}-E(x_{t})\right)}^{2}{\left(x_{t+\tau }-E(x_{t+\tau })\right)}^{2}\right),\;\tau >0,$$

reveal the temporal dependence when not being equal to $E\left({\left(x_{t}-E(x_{t})\right)}^{2}\right)E\left((x_{t+\tau }-\right.$ $\left.E(x_{t+\tau })){}^{2}\right)$. In Section 4 we present an ICA estimator which takes into account both linear and quadratic autocovariances of the components to solve the ICA problem. By doing so, we enable efficient estimation of various kinds of stationary time series. Before that we, however, shortly recall the ARMA‐GARCH processes, which are used to model the time series exhibiting volatility clustering.

## 3 Arma‐Garch Model

Let now $F_{t}$ denote the information of a univariate time series process (*x*_*t*_)_*t* = 0, ± 1, ± 2,…_ at time *t*. The conditional mean process of a time series process

$$x_{t}=E(x_{t}|F_{t-1})+z_{t}$$

is often modelled as an ARMA(*p*,*q*) process

$$E(x_{t}|F_{t-1})=\overset{p}{\underset{i=1}{\sum }}\varphi_{i}x_{t-i}+\overset{q}{\underset{j=1}{\sum }}\theta_{j}z_{t-j},$$

where *φ*_1_,…,*φ*_*p*_ are the autoregressive coefficients and *θ*_1_,…,*θ*_*q*_ are the moving average coefficients. The ARMA process is causal if it can be written as a MA(*∞*) process

$$x_{t}=\overset{\infty }{\underset{i=0}{\sum }}\psi_{i}z_{t-i}.$$

The process (*z*_*t*_)_*t* = 0, ± 1, ± 2,…_ is a white noise process which is often assumed to consist of i.i.d. Gaussian random variables. The ARMA‐GARCH process (see e.g. Weiss (1984), Bollerslev (1986)) is then obtained when *z* is a GARCH(*P*,*Q*) process

$$z_{t}=\sigma_{t}\epsilon_{t},$$

where *ϵ*_*t*_ is a Gaussian white noise process with unit variance and $\sigma_{t}^{2}$ is a conditional variance process

$$\begin{array}{ll} \sigma_{t}^{2} & =\mathrm{var}(z_{t}|F_{t-1})=\omega +\overset{P}{\underset{i=1}{\sum }}\alpha_{i}z_{t-i}^{2}+\overset{Q}{\underset{j=1}{\sum }}\beta_{j}\sigma_{t-j}^{2},\; \end{array}$$

with *ω* > 0 and *α*_*i*_,*β*_*j*_ ≥ 0 for all *i* = 1,…,*P* and *j* = 1,…,*Q*. The process is second‐order stationary if $\sum_{i=1}^{p}\alpha_{i}+\sum_{j=1}^{q}\beta_{j}<1$ (Bollerslev, 1986).

In this article, we focus on *p*‐variate causal ARMA‐GARCH(1,1) processes (***x***_*t*_)_*t* = 0, ± 1, ± 2,…_ with mutually independent components

(1) $$\begin{array}{ll} x_{tj} & =\overset{\infty }{\underset{i=0}{\sum }}\psi_{ij}z_{t-i,j},\;\;j=1,\text{…},p,\; \end{array}$$

where $\sum_{i=0}^{\infty }\psi_{ij}^{2}=1$. The process *z*_*tj*_ is a GARCH(1,1) process

$$\begin{array}{ll} z_{tj} & =\sigma_{tj}\epsilon_{tj},\;\;j=1,\text{…},p,\; \end{array}$$

where *ϵ*_*tj*_ is a Gaussian white noise process with unit variance and

$$\begin{array}{ll} \sigma_{tj}^{2} & =\omega_{j}+\alpha_{j}z_{t-1,j}^{2}+\beta_{j}\sigma_{t-1,j}^{2},\; \end{array}$$

with *α*_*j*_,*β*_*j*_ ≥ 0 and *ω*_*j*_ = 1 − *α*_*j*_ − *β*_*j*_. This implies that var(*z*_*tj*_) = 1 and further var(*x*_*tj*_) = 1. We restrict to processes with finite eighth moments. Notice that a GARCH(1,1) process with parameters *α* and *β* has finite eighth moments if and only if *β* ^4^ + 4*β* ^3^ *α* + 18*β* ^2^ *α* ^2^ + 60*βα* ^3^ + 105*α* ^4^ < 1. For assessing the finiteness of even moments of a GARCH(1,1) process, see Bollerslev (1986).

## 4 Generalized SOBI Estimator

### 4.1 Classical SOBI Estimator

A general idea in ICA is to find for the standardized series $x_{t}^{st}$ the orthogonal transformation, that is, the *p* × *p* rotation matrix ***U*** which maximizes some prechosen objective function. In the following we show how the objective function yielding the classical SOBI estimator (Belouchrani et al. 1997) is obtained. Let $S_{\tau }(x_{t})=E(x_{t}x_{t+\tau }^{T})$ denote the autocovariance matrix with lag *τ* for a centred process ***x***. Then, for a set of lags $T=\{\tau_{1},\text{…},\tau_{K}\}$, the rotation matrix ***U*** in the SOBI estimator is defined as the orthogonal matrix which makes the transformed autocovariance matrices

$$US_{\tau_{1}}(x_{t}^{st})U^{T},\text{…},US_{\tau_{K}}(x_{t}^{st})U^{T}$$

as diagonal as possible, the diagonality being measured using the sum of squares of the off‐diagonal elements. Notice that, since, for each *i*, the sum of squares of all elements of $US_{\tau_{i}}(x_{t}^{st})U^{T}$ remain the same for any orthogonal ***U***, the diagonality can be alternatively measured using the sum of squares of the diagonal elements. Hence, the SOBI unmixing matrix estimator **Γ** can be defined as **Γ**=***US***(***x***_*t*_)^−1/2^, where ***U***=(***u***_1_,…,***u***_2_)^*T*^ is the maximizer of the objective function

$$\overset{p}{\underset{j=1}{\sum }}\overset{K}{\underset{k=1}{\sum }}{\left(u_{j}^{T}S_{\tau_{k}}(x_{t}^{st})u_{j}\right)}^{2}$$

under the orthogonality constraint ***UU*** ^*T*^ = ***I***_*p*_. This is equivalent to finding **Γ**=(***γ***_1_,…,***γ***_*p*_)^*T*^ which maximizes

$$\overset{p}{\underset{j=1}{\sum }}\overset{K}{\underset{k=1}{\sum }}{\left(\gamma_{j}^{T}S_{\tau_{k}}(x_{t})\gamma_{j}\right)}^{2}=\overset{p}{\underset{j=1}{\sum }}\overset{K}{\underset{k=1}{\sum }}{\left(E\left(\gamma_{j}^{T}x_{t}\gamma_{j}^{T}x_{t+\tau_{k}}\right)\right)}^{2},$$

under the constraint **Γ*S***(***x***_*t*_)**Γ** ^*T*^ = ***I***_*p*_. Notice that when *K* = 1 the estimator is known as the algorithm for multiple unknown signals extraction (AMUSE) estimator (Tong et al. 1990) which can be obtained as the solution for an eigenvalue‐eigenvector problem. The use of more autocovariance matrices, as in SOBI, is however preferred as AMUSE depends heavily on the choice of the lag. For general statistical properties of AMUSE and SOBI estimators (especially assuming linear processes), computational issues, choice of lags and variants, see Miettinen et al. (2012), Miettinen et al. (2014), Miettinen et al. (2016), Illner et al. (2015) and Taskinen et al. (2016).

### 4.2 Generalized SOBI Estimator: Estimating Equations

A more flexible use of temporal dependence in ICA has been considered in Hyvärinen (2001), Shi et al. (2009), Matteson and Tsay (2011) and Matilainen et al. (2017), the last of which defined variant of SOBI (vSOBI) estimator as the maximizer of

$$\overset{p}{\underset{j=1}{\sum }}\overset{K}{\underset{k=1}{\sum }}{\left(E\left(G(\gamma_{j}^{T}x_{t})G(\gamma_{j}^{T}x_{t+\tau_{k}})\right)-E{\left(G(\gamma_{j}^{T}x_{t})\right)}^{2}\right)}^{2},$$

under the constraint **Γ*S***(***x***_*t*_)**Γ** ^*T*^ = ***I***_*p*_. Here *G* is a twice continuously differentiable function and the SOBI estimator is naturally obtained by the choice *G*(*x*) = *x*. Matilainen et al. (2017) also discussed the possibility of combining the classical SOBI estimator with the vSOBI estimator given another function *G*. In this article, we study such hybrid estimator in more detail. We choose as a *G* function *G*(*x*) = *x* ^2^ and define the generalized SOBI (gSOBI) estimator as the maximizer of

(2) $$\begin{array}{ll} & b\underset{\tau \in T_{1}}{\sum }\overset{p}{\underset{j=1}{\sum }}{\left(E\left(\gamma_{j}^{T}x_{t}\gamma_{j}^{T}x_{t+\tau })\right)\right)}^{2}\;\\ & \;+(1-b)\underset{\tau \in T_{2}}{\sum }\overset{p}{\underset{j=1}{\sum }}{\left(E\left({\left(\gamma_{j}^{T}x_{t}\right)}^{2}{\left(\gamma_{j}^{T}x_{t+\tau }\right)}^{2}-1\right)\right)}^{2},\;\;0\leq b\leq 1,\; \end{array}$$

under the constraint **Γ*S***(***x***_*t*_)**Γ** ^*T*^ = ***I***_*p*_. Here $T_{1}$ and $T_{2}$ are the sets of lags for the linear and quadratic parts respectively, and *b* is a parameter which gives the weight for the linear part.

The estimating equations for SOBI and vSOBI were derived in Miettinen et al. (2016) and Matilainen et al. (2017) respectively. In a similar fashion, the estimating equations for gSOBI can be derived and they are as follows.

Definition 1 The generalized SOBI unmixing matrix functional **Γ**=(***γ***_1_,…,***γ***_*p*_)^*T*^ solves the estimating equations

$$\begin{array}{ll} & \gamma_{j}^{T}T(\gamma_{l})=\gamma_{l}^{T}T(\gamma_{j})\;\;\text{and}\;\;\gamma_{j}^{T}S\gamma_{l}=\delta_{jl},\;\;j,l=1,\text{…},p,\; \end{array}$$

where *δ*_*jl*_ = 1, if *j* = *l* and 0 otherwise,

$$T(\gamma )=bT^{s}(\gamma )+(1-b)T^{v}(\gamma )$$

and

$$\begin{array}{ll} T^{s}(\gamma )= & \underset{\tau \in T_{1}}{\sum }\left(E\left(\gamma^{T}x_{t}\gamma^{T}x_{t+\tau }\right)E\left(\left(\gamma^{T}x_{t+\tau }\right)x_{t}+\left(\gamma^{T}x_{t}\right)x_{t+\tau }\right)\right),\;\\ T^{v}(\gamma )= & \;2\underset{\tau \in T_{2}}{\sum }\left(E\left({\left(\gamma^{T}x_{t}\right)}^{2}{\left(\gamma^{T}x_{t+\tau }\right)}^{2}-1\right)\right.\;\\ & \left.\;\;\;\times E\left(\left(\gamma^{T}x_{t}\right){\left(\gamma^{T}x_{t+\tau }\right)}^{2}x_{t}+{\left(\gamma^{T}x_{t}\right)}^{2}\left(\gamma^{T}x_{t+\tau }\right)x_{t+\tau }\right)\right).\; \end{array}$$

The corresponding generalized SOBI unmixing estimator $\hat{\Gamma }$ is then obtained by replacing the expected values in the estimating equations by sample means. The estimating equations now allow us to study the asymptotical properties of the gSOBI estimator.

### 4.3 Asymptotic Properties

Due to the affine equivariance of the gSOBI estimator, see Miettinen et al. (2016) and Matilainen et al. (2017), the limiting distribution of $\sqrt{n}\;(\hat{\Gamma }\Omega -I_{p})$ is the same for any mixing matrix **Ω**. Hence, when deriving the asymptotic distribution, we may first assume that **Ω**=***I***_*p*_. The limiting distribution in general case then follows as $\text{vec}(\hat{\Gamma }\Omega )=(\Omega^{T}⊗I_{p})\text{vec}(\hat{\Gamma })$. Here vec is an operator which vectorizes a matrix by stacking its columns on top of each other.

Let now ***e***_*j*_ denote a *p*‐vector whose *j*th element equals to one and the others are zeros. We then write $T_{j}=bT_{j}^{s}+(1-b)T_{j}^{v}$, where

$$\begin{array}{ll} T_{j}^{s}= & \underset{\tau \in T_{1}}{\sum }\left(\overset{n-\tau }{\underset{t=1}{\sum }}\left(e_{j}^{T}x_{t}e_{j}^{T}x_{t+\tau }\right)\overset{n-\tau }{\underset{t=1}{\sum }}\left(\left(e_{j}^{T}x_{t}\right)x_{t+\tau }+\left(e_{j}^{T}x_{t+\tau }\right)x_{t}\right)\right),\;\\ T_{j}^{v}= & \;2\underset{\tau \in T_{2}}{\sum }\left(\overset{n-\tau }{\underset{t=1}{\sum }}\left({\left(e_{j}^{T}x_{t}\right)}^{2}{\left(e_{j}^{T}x_{t+\tau }\right)}^{2}-1\right))\right.\;\\ & \left.\;\;\;\times \overset{n-\tau }{\underset{t=1}{\sum }}\left(\left(e_{j}^{T}x_{t}\right){\left(e_{j}^{T}x_{t+\tau }\right)}^{2}x_{t}+{\left(e_{j}^{T}x_{t}\right)}^{2}\left(e_{j}^{T}x_{t+\tau }\right)x_{t+\tau }\right)\right).\; \end{array}$$

For our main theorem (Theorem 1), we make the following three assumptions on the independent components ***s***.

(A1) E(***s***)=**0** and cov(***s***) = ***I***_*p*_.
(A2) The *p* time series in ***s*** are independent.
(A3) For each pair *j* ≠ *l* of independent components
  (a) there exists a $\tau \in T_{1}$ such that *μ*_*τj*_ ≠ *μ*_*τl*_, or
  (b) there exists a $\tau \in T_{2}$ such that $\nu_{\tau j}^{2}+\nu_{\tau l}^{2}-2(\nu_{\tau j}+\nu_{\tau l})\mu_{\tau j}\mu_{\tau_{l}}\neq 0$,
  where *μ*_*τj*_ = E(*s*_*tj*_ *s*_*t* + *τ*,*j*_) and $\nu_{\tau j}=E(s_{tj}^{2}s_{t+\tau ,j}^{2})-1$.

The assumption (A3) is sufficient as such when *b* < 1, and part (a) is required for the case *b* = 1. For an ARMA model it can be shown that $\nu_{\tau j}=2\mu_{\tau j}^{2}$, and therefore, if *μ*_*τj*_ = *μ*_*τl*_ for all $\tau \in T_{2}$, part (b) of (A3) is not satisfied. Hence, identical ARMA processes without stochastic volatility cannot be separated. On the other hand, identical (ARMA‐)GARCH processes as well as mutually dissimilar processes can be separated. Theorem 1 and Corollary 1 now generalize the results for ARMA model (Miettinen et al. 2016) to the more general ARMA‐GARCH model. Notice that the results for *b* ≠ 1 have not been derived before for any time series model. The following results are proved in the Supporting information.

Theorem 1 Under the assumptions (A1)–(A3) and **Ω**=***I***_*p*_, we have that $\hat{\Gamma }\to_{P}I_{p}$ and

$$\begin{array}{ll} & \sqrt{n}\;({\hat{\gamma }}_{jj}-1)=-\frac{1}{2}\sqrt{n}({\hat{S}}_{jj}-1)+o_{p}(1),\;\\ & \sqrt{n}\;{\hat{\gamma }}_{jl}=\left(e_{l}^{T}\sqrt{n}\;T_{j}-e_{j}^{T}\sqrt{n}\;T_{l}+\left(2b\underset{\tau \in T_{1}}{\sum }\mu_{\tau j}(\mu_{\tau l}-\mu_{\tau j})\right.\right.\;\\ & \left.\;\;\;\;+\left.4(1-b)\underset{\tau \in T_{2}}{\sum }\left(\nu_{\tau l}-\nu_{\tau j}(\nu_{\tau j}+1)+2\nu_{\tau l}\mu_{\tau j}\mu_{\tau l}\right)\right)\sqrt{n}\;{\hat{S}}_{jl}\right)\;\\ & \;\;\;\;\times {\left(2b\underset{\tau \in T_{1}}{\sum }{\left(\mu_{\tau j}-\mu_{\tau l}\right)}^{2}+4(1-b)\underset{\tau \in T_{2}}{\sum }\left(\nu_{\tau j}^{2}+\nu_{\tau l}^{2}-2(\nu_{\tau j}+\nu_{\tau l})\mu_{\tau j}\mu_{\tau_{l}}\right)\right)}^{-1}\;\\ & \;\;\;\;+o_{p}(1),\;\;\;j\neq l,\; \end{array}$$

where $\hat{S}$ is the sample covariance matrix.

From Theorem 1 we obtain a straightforward corollary.

Corollary 1 In addition to (A1)–(A3), assume that ***s*** has finite eighth moments, and that the elements of $\hat{S}$, $T_{j}^{s}$, and $T_{j}^{v}$, *j* = 1,…,*p* are asymptotically normally distributed. Then the limiting distribution of $\text{vec}(\hat{\Gamma }-I_{p})$ is normal with zero mean and the following variances.

$$\begin{array}{ll} ASV({\hat{\gamma }}_{jj}) & =4^{-1}\left(E\left[x_{tj}^{4}\right]+\overset{\infty }{\underset{k=-\infty }{\sum }}\left(E\left[x_{tj}^{2}x_{t+k,j}^{2}\right]-1\right)-1\right)\;\\ ASV\left({\hat{\gamma }}_{jl}\right) & =\left(b^{2}ASV\left(e_{l}^{T}T_{j}^{s}\right)+2b(1-b)ASCOV\left(e_{l}^{T}T_{j}^{s},e_{l}^{T}T_{j}^{v}\right)\right.\;\\ & \;+{\left(1-b\right)}^{2}ASV\left(e_{l}^{T}T_{j}^{v}\right)+b^{2}ASV\left(e_{j}^{T}T_{l}^{s}\right)+2b(1-b)ASCOV\left(e_{j}^{T}T_{l}^{s},e_{j}^{T}T_{l}^{v}\right)\;\\ & \;+{\left(1-b\right)}^{2}ASV\left(e_{j}^{T}T_{l}^{v}\right)-2b^{2}ASCOV\left(e_{l}^{T}T_{j}^{s},e_{j}^{T}T_{l}^{s}\right)-2{\left(1-b\right)}^{2}\;\\ & \;\times ASCOV\left(e_{l}^{T}T_{j}^{v},e_{j}^{T}T_{l}^{v}\right)-2b(1-b)\left(ASCOV\left(e_{l}^{T}T_{j}^{s},e_{j}^{T}T_{l}^{v}\right)\right.\;\\ & \left.\;+ASCOV\left(e_{j}^{T}T_{l}^{s},e_{l}^{T}T_{j}^{v}\right)\right)+2c_{jl}\left(b\;ASCOV\left(e_{l}^{T}T_{j}^{s},{\hat{S}}_{jl}\right)+(1-b)\right.\;\\ & \;\times ASCOV\left(e_{l}^{T}T_{j}^{v},{\hat{S}}_{jl}\right)-b\;ASCOV\left(e_{j}^{T}T_{l}^{s},{\hat{S}}_{jl}\right)-(1-b)\;\\ & \;\times \left.\left.ASCOV\left(e_{j}^{T}T_{l}^{v},{\hat{S}}_{jl}\right)\right)+c_{jl}^{2}ASV\left({\hat{S}}_{jl}\right)\right)\times \left(2b\underset{\tau \in T_{1}}{\sum }{\left(\mu_{\tau j}-\mu_{\tau l}\right)}^{2}\right.\;\\ & {\left.\;+4(1-b)\underset{\tau \in T_{2}}{\sum }\left(\nu_{\tau j}^{2}+\nu_{\tau l}^{2}-2(\nu_{\tau j}+\nu_{\tau l})\mu_{\tau j}\mu_{\tau l}\right)\right)}^{-2},\;\;\;j\neq l,\; \end{array}$$

where

$$c_{jl}=4(1-b)\underset{\tau \in T_{2}}{\sum }\left(\nu_{\tau l}-\nu_{\tau j}(\nu_{\tau j}+1)+2\nu_{\tau l}\mu_{\tau j}\mu_{\tau l}\right)+2b\underset{\tau \in T_{1}}{\sum }\left(\mu_{\tau j}(\mu_{\tau l}-\mu_{\tau j})\right).$$

If the components of ***s*** are ARMA‐GARCH processes with finite eighth moments, the assumptions of Corollary 1 are satisfied. In the Supporting information, we provide expression for the variances in the case of ARMA‐GARCH(1,1) processes. In Section 6.3 we use the asymptotic results as well as simulation studies to compare the properties of gSOBI estimators with different values of *b*.

### 4.4 Tests for Linear and Quadratic Autocorrelations

Hu and Tsay (2014) argued that especially in the context of econometric time series the latent series that exhibit ‘more’ stochastic volatility are the most interesting ones. An ordering of components according to the ‘degree’ of volatility clustering inherent in the components is therefore an important task and will be considered next. We also demonstrate how to identify those components which do not contain such volatility clustering.

We suggest the following procedure for ordering the estimated components. Notice first that the straightforward use of

(3) $$\begin{array}{ll} \underset{\tau \in T_{2}}{\sum }{\left(\frac{\overset{n-\tau }{\underset{t=1}{\sum }}\left({ŝ}_{tj}^{2}{ŝ}_{t+\tau ,j}^{2}\right)}{(n-\tau )-1}\right)}^{2}, & \; \end{array}$$

as a criterion for ordering does not work, since linear autocorrelations imply quadratic autocorrelations. Therefore an ARMA model should first be fitted to each time series exhibiting linear autocorrelation, after which the residual series can be plugged into (3) for ordering the components.

To test whether fitting an ARMA model is needed, we propose a modification of the Ljung–Box test, where the asymptotic variance of the test statistic is derived when assuming only symmetry and finite fourth moments of the time series.

Definition 2 The modified Ljung–Box test statistic using a set of lags T is given by

$$L=n\underset{\tau \in T}{\sum }\frac{{\left(\overset{n-\tau }{\underset{t=1}{\sum }}\frac{x_{t}x_{t+\tau }}{n-\tau }\right)}^{2}}{V_{\tau }},$$

where *x* is standardized to have zero mean and unit variance, and

$$V_{\tau }=\overset{n-\tau }{\underset{t=1}{\sum }}\frac{x_{t}^{2}x_{t+\tau }^{2}}{n-\tau }+2\overset{n-\tau -1}{\underset{k=1}{\sum }}\frac{n-k}{n}\overset{n-k-\tau }{\underset{t=1}{\sum }}\frac{x_{t}x_{t+\tau }x_{t+k}x_{t+k+\tau }}{n-k-\tau }.$$

Notice that, when *x*_1_,…,*x*_*n*_ are i.i.d., *V*_*τ*_→_*P*_1 and the classical Ljung–Box test is obtained by *V*_*τ*_ = 1. However, in the case of *x* having volatility clustering, *V*_*τ*_ > 1, and the estimation of *V*_*τ*_ is required to achieve the correct size for the test.

Theorem 2 Under the null hypothesis that E(*x*_*t*_ *x*_*t* + *τ*_) = 0 for all *τ* = 1,2,…, the limiting distribution of *L* is the chi‐squared distribution with |T| degrees of freedom.

Since E(*x*_*t*_ *x*_*t* + *τ*_ *x*_*t* + *k*_ *x*_*t* + *k* + *τ*_) is typically very close to zero for large *k*, we compute in our simulation studies *V*_*τ*_ using *k* = 1,…,20.

To test whether the quadratic autocorrelations are typical for homoscedastic time series, we use the following test statistic.

Definition 3 The test statistic for volatility clustering, using a set of lags T, is

$$Q=n\underset{\tau \in T}{\sum }{\left(\overset{n-\tau }{\underset{t=1}{\sum }}\frac{x_{t}^{2}x_{t+\tau }^{2}}{n-\tau }-1\right)}^{2}/4,$$

where *x* is standardized to have zero mean and unit variance.

Before using the test statistic *Q*, one should remove the linear autocorrelation from the series. This can be performed, for example, by fitting an ARMA model and then applying the test statistic to the residual series.

Theorem 3 Under the null hypothesis that *x*_1_,…,*x*_*n*_ are i.i.d. Gaussian, the distribution of *Q* is the chi‐squared distribution with |T| degrees of freedom.

The validity of the test statistics *L* and *Q* under finite sample sizes is studied in Section 6.4.

## 5 PVC Analysis and ICA

In Section 6 we compare the asymptotic and finite sample properties of different gSOBI estimators. Interestingly, we can also include a slight modification of the PVC estimator (Hu and Tsay, 2014) in the comparisons as it solves the ICA problem as shown in the following. PVC and vSOBI are similar in the sense that both of them use fourth‐order moments of the form $E(x_{t}^{2}x_{t+\tau }^{2})$. The differences of the methods are discussed in the end of this section.

The lag‐*τ* generalized kurtosis matrix of a *p*‐variate time series ***x***_*t*_ was defined in Hu and Tsay (2014) as

$$g_{\tau }(x_{t})=\overset{p}{\underset{i=1}{\sum }}\overset{p}{\underset{j=i}{\sum }}{\left(\text{cov}(x_{t}x_{t}^{T},y_{t-\tau ,ij})\right)}^{2},$$

where *y*_*t* − *τ*,*ij*_ is a function of *x*_*t* − *τ*,*i*_ *x*_*t* − *τ*,*j*_ for 1 ≤ *i*,*j* ≤ *p* and *τ* ≥ 0. For what follows, we restrict ourselves to the identity function *y*_*t* − *τ*,*ij*_ = *x*_*t* − *τ*,*i*_ *x*_*t* − *τ*,*j*_, and run the index *j* from 1 to *p*, that is, we define

$$g_{\tau }(x_{t})=\overset{p}{\underset{i=1}{\sum }}\overset{p}{\underset{j=1}{\sum }}{\left(\text{cov}(x_{t}x_{t}^{T},x_{t-\tau ,i}x_{t-\tau ,j})\right)}^{2}.$$

Notice that this definition is slightly different from the one originally suggested in Hu and Tsay (2014), where the second index runs from *i* to *p*. Affine equivariance of the ICA unmixing matrix, as proven below, is however only achievable with this modification.

The cumulative generalized kurtosis matrix is now given by

$$G_{m}(x_{t})=\overset{m}{\underset{\tau =1}{\sum }}g_{\tau }(x_{t}).$$

Theorem 4 states that this version of ***G***_*m*_ possesses the two properties which are required when solving the ICA problem, see Oja et al. (2006) and Nordhausen and Tyler (2015). The proofs are given in the Supporting information.

Theorem 4

(i) ***G***_*m*_ is orthogonal equivariant, that is, for any *p*‐variate random vector ***x*** and any *p* × *p* orthogonal matrix ***V***,
  $$G_{m}(Vx_{t})=VG_{m}(x_{t})V^{T}.$$
(ii) ***G***_*m*_ has the independence property, that is, if ***s*** has independent components, then ***G***_*m*_(***s***_*t*_) is diagonal.

Hence, we may construct the PVC estimator for the independent component problem as a simultaneous diagonalizer of the covariance matrix ***S*** and the cumulative generalized kurtosis matrix ***G***_*m*_.

Definition 4 The PVC unmixing matrix functional **Γ** satisfies

$$\Gamma S(x_{t})\Gamma^{T}=I_{p}\;\text{and}\;\Gamma G_{m}(x_{t})\Gamma^{T}=D,$$

where ***D*** is a diagonal matrix.

In practice, the estimator can thus be obtained by first whitening the times series using the covariance matrix ***S*** and then by finding the eigendecomposition of ***G***_*m*_(***x*** ^*st*^). The resulting unmixing matrix functional is affine equivariant as one of the two matrices is affine equivariant and the other one is orthogonal equivariant (Oja et al. 2006).

Simultaneous diagonalization separates a pair of independent components only if the corresponding eigenvalues are distinct. Therefore, in the case of PVC we have to assume that, for *i* ≠ *j*,

$$\overset{m}{\underset{l=1}{\sum }}{\left(E((s_{ti}^{2}-1)(s_{t-l,i}^{2}-1))\right)}^{2}\neq \overset{m}{\underset{l=1}{\sum }}{\left(E((s_{tj}^{2}-1)(s_{t-l,j}^{2}-1))\right)}^{2}.$$

This is stronger than the assumption that vSOBI needs, that is, for each component *i* = 1,…,*p*, there is at least one lag *τ* such that $E(s_{ti}^{2}s_{t+\tau ,i}^{2})\neq 1$. Notice that the assumptions differ even if only one lag is used, as PVC requires that the values are distinct for different independent components, but vSOBI does not. There is also a fundamental difference in how multiple lags are exploited. In PVC, the matrices given by different lags are summed into a single matrix and the unmixing matrix is derived using that. In vSOBI the different lags contribute to the unmixing matrix independently and then the contributions are summed. The simultaneous diagonalization of two matrices is a computationally simple method to solve the independent component problem, but the estimation efficiency is known to be inferior to more advanced methods.

## 6 Simulation Study

We compare the performances of different gSOBI estimators to that of the PVC estimator. The vSOBI estimator was compared to some other methods in Matilainen et al. (2017). All computations have been performed in R software (R Core Team, 2017) using the packages JADE (Miettinen et al. 2017), tsBSS (Matilainen et al. 2018), forecast (Hyndman, 2017) and fGarch (Wuertz and Rmetrics Core Team, 2013).

### 6.1 Performance Index

To measure the performances of the estimates in the simulation study, we use the minimum distance index (MDI, Ilmonen et al., 2010) defined as

$$\begin{array}{ll} \hat{D}=D(\hat{\Gamma }\Omega )=\frac{1}{\sqrt{p-1}}\underset{C\in C}{\inf}\|C\hat{\Gamma }\Omega -I_{p}\|, & \; \end{array}$$

where ‖·‖ is the matrix (Frobenius) norm and

C={C:each row and column ofChas exactly one non‐zero element}.

The index takes values between zero and one, zero indicating perfect separation. The MDI is simple to compute and it has some attractive properties. It is invariant with respect to the change of the mixing matrix, and when the estimate $\hat{\Gamma }$ is asymptotically normally distributed with expected value ***I***_*p*_, then the limiting expected value of $n(p-1){\hat{D}}^{2}$ is the sum of the limiting variances of the off‐diagonal elements of $\sqrt{n}\;\hat{\Gamma }$. As the sum provides a global measure of the variation, the limiting expected values of $n(p-1){\hat{D}}^{2}$ can be compared for asymptotical efficiencies (Ilmonen et al. 2010).

### 6.2 Models

We consider four different time series models in our performance comparisons. First, model (i) consists of a three‐variate ARMA(1,1)‐GARCH(1,1) process with the parameter values given in Table I. Model (ii) is a three‐variate pure ARMA(1,1) process and model (iii) is a three‐variate pure GARCH(1,1) process, where *φ* and *θ* of model (ii) and *α*, *β* and *ω* of model (iii) take values as in Table I. In model (iv) there are two pure ARMA(1,1) components and two pure GARCH(1,1) components with parameters as in *s*_1_ and *s*_2_ in Table I. Since all the estimators included in comparisons are affine equivariant, we use **Ω**=***I***_*p*_ as a mixing matrix.

**Table I:** The parameter values of the ARMA‐GARCH time series in model (i). Parameter *φ* is the AR coefficient and *θ* is the MA coefficient

	*α*	*β*	*ω*	*φ*	*θ*
*s*_1_	0.15	0.7	0.15	0.5	‐0.1
*s*_2_	0.1	0.8	0.1	0.2	0.8
*s*_3_	0.05	0.9	0.05	0.1	0.1

### 6.3 Efficiency Results

**Figure 1:**
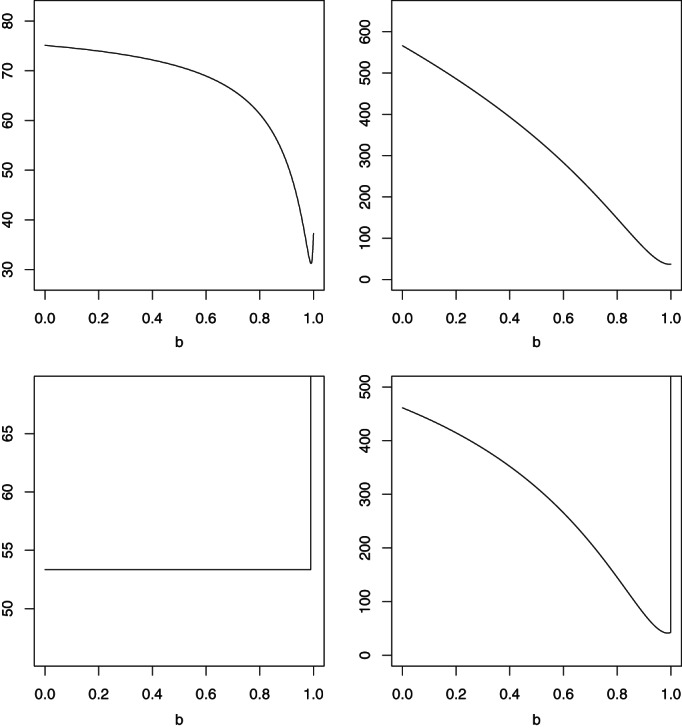
The limiting expected value of $n(p-1){\hat{D}}^{2}$ as function of *b* in model (i) on the top left, (ii) on the top right, (iii) on the bottom left and (iv) on the bottom right.

To guide the selection of *b* in gSOBI estimator we first compare the asymptotic efficiencies of estimators as a function of *b*. In our simulations, we selected the lag sets $T_{1}=\left\{1,2,3\right\}$ and $T_{2}=\left\{1,2,3\right\}$, but used also the lag set ${\tilde{T}}_{1}=\left\{1,\text{…},12\right\}$ for *b* = 0.9. The limiting expected values of $n(p-1){\hat{D}}^{2}$ as a function of *b* for models (i)–(iv) are plotted in Figure 1. As seen in the figure, in model (iii), that is, the model with three GARCH(1,1) processes, the expected value is constant for *b* < 1 and goes to infinity when *b* = 1. For other models, large values of *b* are preferable excluding SOBI (*b* = 1) which totally fails in models (iii) and (iv), that is, in models with more than one component with zero linear autocorrelation for all lags.

**Figure 2:**
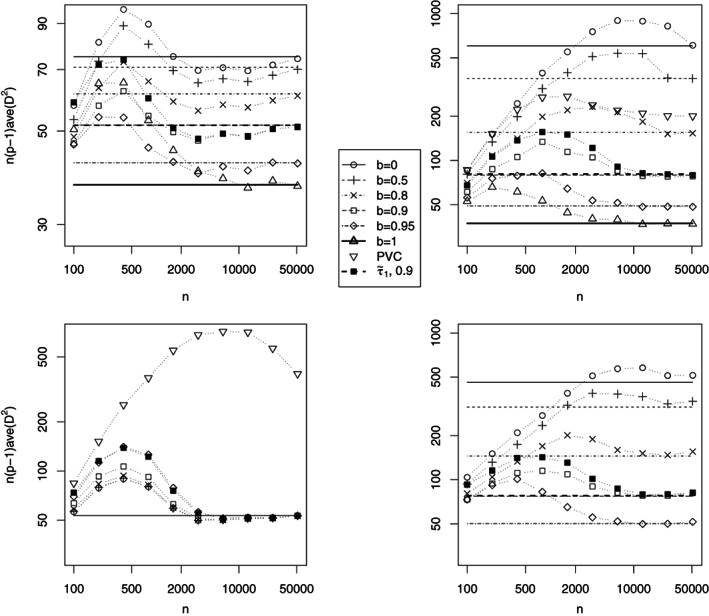
Simulation results for models (i)–(iv). The points show the averages of $n(p-1){\hat{D}}^{2}$ over 2000 repetitions for each sample size, and the horizontal lines give the asymptotic expected values. PVC is out of the plotting region for models (i) and (iv) and SOBI (*b* = 1) for models (iii) and (iv), and therefore not visible. Results are shown for model (i) on the top left, (ii) on the top right, (iii) on the bottom left and (iv) on the bottom right respectively. Both axes are plotted on log scales.

The finite sample behaviour of six gSOBI estimators given by *b* = 0, *b* = 0.5, *b* = 0.8, *b* = 0.9, *b* = 0.95 and *b* = 1, and the PVC estimator with *m* = 5 is compared using the following simulation study. For sample sizes *n* = 100,200,400,…,51200 we generated 2000 realizations from models (i) to (iv). Figure 2 shows the averages of $n(p-1){\hat{D}}^{2}$ over the 2000 repetitions. As expected, SOBI (*b* = 1) performs poorly in models (iii) and (iv), and is therefore omitted from those figures. PVC is dropped out of the figures corresponding to models (i) and (iv) as the averages of $n(p-1){\hat{D}}^{2}$ grow with *n*. In model (i), the performance of PVC can be improved by following the recommendation given in the application section of Hu and Tsay (2014), to apply PVC to the residuals of vector autoregressive (VAR) model, and the results are even better if the data is standardized before finding the VAR residuals, not after it. Then the results of PVC are similar to those in model (iii). On the other hand, in models (ii) and (iv), the effect of using residuals would be negative and in model (iii) it would be neutral. In model (iii), all gSOBI estimators with *b* < 1 are asymptotically equally efficient, and in the simulations they are practically equally good from *n* = 3200 on outperforming PVC at all sample sizes. With smaller sample sizes the unnecessary use of linear autocorrelations causes loss in efficiency. However, the estimator with *b* = 0.5 is already as good as the one with *b* = 0. In model (ii) with only ARMA components, the quadratic autocorrelations are non‐zero and therefore the choice *b* = 0 also works. However, the estimators with *b* closer to 1 perform much better. All in all, it seems clear that for separating various kinds of stationary time series, estimators with 0 < *b* < 1 perform better than those with *b* = 0 and *b* = 1, and that *b* should be larger than 0.5. We thus suggest the use of value *b* = 0.9. The lag set $T_{1}$ is here slightly better than ${\tilde{T}}_{1}$, but the latter one is generally a safer choice.

### 6.4 Tests

To evaluate the performances of the test statistics proposed in Section 4.4, for each data set generated according to models (i), (ii) and (iii), the tests for linear and quadratic autocorrelations were performed. In the tests, we used the estimates of the independent component time series given by gSOBI with *b* = 0.9. Table II presents the proportions of rejected null hypotheses at significance level 0.05, when testing the linear autocorrelations in models (i), (ii) and (iii), and Table III presents the proportions of rejected null hypotheses in the quadratic autocorrelations test. When testing for linear autocorrelations, both the modified and the classical Ljung–Box tests were applied. The results in Table II show that in the GARCH model (iii), where the linear autocorrelations are zero, the modified Ljung–Box test has the correct rate of false rejections, whereas the classical test rejects the null hypothesis too often. Recall that the test for quadratic autocorrelations is applied to residual series of ARMA models, and that the estimated components are ordered according to the values of the test statistic. Therefore, especially in the case of model (ii), we are mainly interested in the sum of the rejection probabilities, which should be 0.15 when there is no volatility clustering in the components. Table III shows that the rate of false rejections is close to the desired level from *n* = 800 upwards.

**Table II:** The proportion of rejections when testing for linear autocorrelations at significance level 0.05 of components *s*_1_, *s*_2_ and *s*_3_ in models (i), (ii) and (iii). The modified Ljung–Box on the left and the classical Ljung–Box on the right

Model	*n*	*s*_1_	*s*_2_	*s*_3_
	100	0.768/0.839	0.874/0.898	0.486/0.523
	200	0.927/0.945	0.948/0.962	0.622/0.665
(i)	400	0.988/0.994	0.988/0.994	0.893/0.925
	800	1/1	0.999/1	0.995/0.998
	≥1600	1/1	1/1	1/1
	100	0.833/0.848	0.847/0.867	0.528/0.545
	200	0.921/0.920	0.919/0.926	0.690/0.696
(ii)	400	0.983/0.986	0.980/0.981	0.933/0.934
	800	1/1	1/1	0.997/0.997
	≥1600	1/1	1/1	1/1
	100	0.122/0.203	0.127/0.184	0.139/0.178
	200	0.094/0.181	0.111/0.175	0.097/0.139
	400	0.068/0.158	0.089/0.142	0.096/0.130
	800	0.059/0.156	0.064/0.134	0.065/0.094
(iii)	1600	0.053/0.147	0.063/0.121	0.049/0.080
	3200	0.052/0.148	0.056/0.118	0.054/0.086
	6400	0.055/0.153	0.048/0.108	0.060/0.087
	12800	0.056/0.153	0.054/0.120	0.055/0.092
	25600	0.048/0.150	0.053/0.119	0.052/0.082
	51200	0.056/0.144	0.046/0.106	0.049/0.072

**Table III:** The proportion of rejections when testing for quadratic autocorrelations at 0.05 significance level of components *s*_1_, *s*_2_ and *s*_3_ in models (i), (ii) and (iii)

Model		(i)			(ii)			(iii)	
*n*	*s*_1_	*s*_2_	*s*_3_	*s*_1_	*s*_2_	*s*_3_	*s*_1_	*s*_2_	*s*_3_
100	0.292	0.180	0.155	0.073	0.075	0.077	0.443	0.308	0.242
200	0.526	0.322	0.258	0.067	0.063	0.062	0.669	0.500	0.336
400	0.841	0.596	0.361	0.062	0.064	0.059	0.878	0.715	0.440
800	0.983	0.911	0.598	0.058	0.049	0.055	0.987	0.929	0.658
1600	1	0.996	0.880	0.056	0.049	0.058	1	1	0.880
3200	1	1	0.996	0.044	0.044	0.055	1	1	0.996
6400	1	1	1	0.053	0.054	0.048	1	1	1
12,800	1	1	1	0.050	0.053	0.051	1	1	1
25,600	1	1	1	0.054	0.050	0.049	1	1	1
51,200	1	1	1	0.041	0.052	0.051	1	1	1

Finally, we examimed for models (i) and (iii) the ordering the components according to volatility

$$\underset{\tau \in T_{2}}{\sum }{\left(\overset{n-\tau }{\underset{t=1}{\sum }}\left(r_{tj}^{2}r_{t+\tau ,j}^{2}\right)/(n-\tau )-1\right)}^{2},$$

where *r* denotes the residual time series of the independent components after fitting the ARMA model. The expected values of volatilities for the three components were 1.88, 1.69 and 0.53. Table IV shows how often the components are ordered correctly at different sample sizes. In the GARCH model (iii), the order is quite consistently the expected one already at small values of *n*, whereas in the ARMA‐GARCH model (i), larger sample size is needed for correct ordering.

**Table IV:** The proportions of correct ordering of the components in models (i) and (iii).

*n*	100	200	400	800	1600	3200	6400	12,800	25,600	51,200
(i)	0.20	0.29	0.40	0.50	0.66	0.79	0.89	0.96	0.99	1.00
(iii)	0.72	0.284	0.95	0.99	1.00	1.00	1.00	1.00	1.00	1.00

## 7 Application

We apply our proposed gSOBI method to weekly log returns of seven different exchange rates against the US dollar. We use the same data as in Hu and Tsay (2014) and compare our results to those derived from their PVC analysis.

The seven exchange rates in the original data, obtained from the database of International Monetary Fund (2017), are those of Australian Dollar (AUD), Canadian Dollar (CAD), Singaporean Dollar (SGD), British Pound (GBP), Swiss Franc (CHF), Norwegian Kroner (NOK) and Swedish Kroner (SEK). The data included are from March 11, 2000 to November 26, 2011 and are available in the R package tsBSS (Matilainen et al. 2018).

Let ***y***_*t*_ be a vector of average values of the exchange rates against US dollar for week *t*. As observed values for gSOBI algorithm we use the logarithmic returns of weekly averages, that is, values ***x***_*t*_ = log(***y***_*t*_) − log(***y***_*t* − 1_), *t* = 1,2,…,*T*. The total length of the series is *T* = 605.

**Figure 3:**
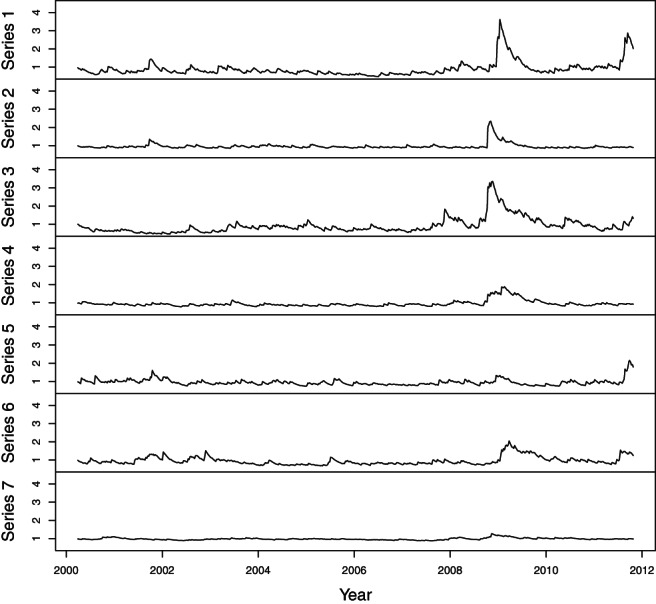
Estimated volatility components of ***s***_*t*_ based on the exchange rate data.

First, we extract the mutually independent components ***s***_*t*_ using gSOBI algorithm with *b* = 0.9, as recommended earlier, and $T_{1}=\{1,\text{…},12\}$ and $T_{2}=\{1,2,3\}$. We then fit stationary and non‐seasonal ARMA(*p*,*q*) models to each series for which the test statistic *L* indicates the presence of linear autocorrelation. Here *p* and *q* are determined using AIC. After removing linear autocorrelations, the residuals of the ARMA fit are used in further analysis to detect the volatility clustering. For each residual series we test for the hypothesis of quadratic autocorrelations being zero using the test statistic *Q*. High value of a quadratic autocorrelation indicates that the volatility changes in time. Components are then ordered according to the degree of volatility clustering, that is, according to the value of *Q*. As in Hu and Tsay (2014), we fit univariate GARCH(1,1) models for the independent component series and plot in Figure 3 the volatility series given by the fitted models.

Table V summarizes the *L*‐ and *Q*‐statistics and corresponding *p*‐values for each independent component based on lags 1,…,5. Also possible ARMA(*p*,*q*) and GARCH(1,1) fits for each relevant component are included. The use of other lag sets in gSOBI estimator or when computing *L*‐ and *Q*‐statistics does not change the results significantly. Most of the independent components have non‐zero linear autocorrelation, and all of the components have statistically significant *Q*‐statistic value. However, the *Q*‐statistic value of the seventh series is much smaller than those of other series, and it can be seen also in Figure 3 that the seventh series has very little volatility clustering as compared to the other series, which have peaks of different magnitudes in volatility series, especially during the years 2008 and 2009.

**Table V:** Values of *L* and *Q*‐statistics with their significance level as well as parameter estimates based on ARMA(*p*,*q*) fit, when applicable, and GARCH(1,1) fits for each logarithmic return series. *Q*‐statistics with superscript ^∗^ means that the test is performed on the residual series

	*s*_1_	*s*_2_	*s*_3_	*s*_4_	*s*_5_	*s*_6_	*s*_7_
*L*	14.33	8.09	6.01	15.19	21.14	13.01	23.79
*p*‐Value	0.014	0.152	0.305	0.010	< 0.001	0.023	< 0.001
ARMA(*p*,*q*):
*φ*_1_	–	–	–	–	‐0.465	0.734	–
*θ*_1_	0.271	–	–	0.187	0.668	‐0.610	0.199
*θ*_2_	–	–	–	–	–	‐0.190	–
*Q*	7418.7^∗^	5242.1	4625.1	464.6^∗^	432.9^∗^	276.6^∗^	18.1^∗^
*p*‐Value	< 0.001	< 0.001	< 0.001	< 0.001	< 0.001	< 0.001	0.0029
GARCH(1,1):
*ω*	0.023	0.120	0.017	0.041	0.076	0.039	0.039
*α*	0.136	0.051	0.141	0.050	0.087	0.086	0.021
*β*	0.849	0.820	0.847	0.905	0.833	0.875	0.938

When comparing the analysis presented above to the one given in Hu and Tsay (2014), there are two key differences. First, Hu and Tsay (2014) start the analysis by fitting a VAR model to remove linear autocorrelations and then extract the PVCs using the residuals of the model. Second, the PVCs are ordered using criteria, which are related to estimation methods. Notice also that the eigenvectors in Hu and Tsay (2014) are not orthogonal, which means that the generalized kurtosis matrix is not symmetrized. This implies that, unlike in most of the ICA methods, the PVCs are not uncorrelated. When comparing the plotted volatility components, we see that the series with most volatility clustering in our analysis, that is, the first and the second series, have similar patterns to series *v*1 and *v*3 in Hu and Tsay (2014). In the other end, both analyses found one component with much less volatility changes than in the other components. In our analysis, the *Q*‐statistics for testing volatility clustering was statistically significant, whereas Hu and Tsay (2014) found out that the component has no volatility clustering.

We also estimated the independent components using other values of *b* in gSOBI. As measures of similarity for a pair of *b* values, we use the average and minimum of the absolute values of the seven correlation coefficients between the estimated independent components, when the independent components are matched so that the average is maximized. Table VI shows how similar the estimates of the independent components are for different values of *b* between 0 and 0.98, even though the results of SOBI and vSOBI differ evidently.

**Table VI:** The average (upper triangle) and minimum (lower triangle) of the matched correlations between the estimates of the independent components

*b*	0	0.9	0.95	0.98	0.99	1
0	1	1.000	0.998	0.975	0.933	0.658
0.9	0.999	1	0.999	0.980	0.940	0.667
0.95	0.995	0.998	1	0.985	0.950	0.678
0.98	0.924	0.936	0.952	1	0.986	0.721
0.99	0.785	0.810	0.840	0.961	1	0.757
1	0.490	0.491	0.491	0.494	0.537	1

This behaviour resembles model (iii) in Section 6, where the quadratic autocorrelations are much larger than the linear autocorrelations.

## 8 Conclusions

The use of both linear and quadratic autocorrelations in ICA enables efficient estimation of different types of latent time series. In this article, we investigated the ICA estimator which is a weighted sum of the classical SOBI estimator (which uses linear autocorrelations) and the recently introduced vSOBI estimator (which uses quadratic autocorrelations). The asymptotic distribution of the resulting generalized SOBI (gSOBI) estimator was derived, and the asymptotic variances were computed using four ARMA‐GARCH models. The gSOBI estimator was shown to outperform its two components, SOBI and vSOBI, especially when some, but not all of the components have non‐zero linear autocorrelations. Even if all the linear autocorrelations are zero, gSOBI with any weighting is asymptotically equally efficient as vSOBI. The choice of weight parameter is crucial for the performance of the gSOBI estimator. The asymptotic variances derived in this article would naturally allow us to choose such weight parameter (from a set of parameter values) that would give the most efficient estimator for the data at hand. Computationally such approach would, however, be too intensive, therefore we can only give recommendations for the weight parameter values based on the simulation studies run in this article. Equal weights for the linear and quadratic parts are not recommended, because then the quadratic part would dominate. Instead, based on our simulation studies, we recommend the use of value *b* = 0.9.

In addition to the asymptotic properties and efficiency comparisons, we also derived new tests for checking whether linear or quadratic autocorrelations are zeros. The test statistic for linear autocorrelations is the same as in the well‐known Ljung–Box test, but we consider the distribution of the test statistic under the null hypothesis of linear independence instead of full independence. Simulation studies indicated that the sizes of the proposed tests are close to the designated one when the sample size is around one thousand or higher.

## Supporting Information

Additional Supporting Information may be found online in the supporting information tab for this article.

## Supporting information

Supporting Information

## Data Availability

The data set used in Section 7 is freely available under the name WeeklyReturnsData in the R package tsBSS which can be downloaded from CRAN (https://cran.r-project.org/package=tsBSS).
