# Supplementary material for: Extracting Conditionally Heteroskedastic Components using Independent Component Analysis
Source: J Time Ser Anal. 2019 Sep 8;41(2):293–311. doi: 10.1111/jtsa.12505 (PMC7266430; doi:10.1111/jtsa.12505)
Supplement: Supplementary file 1 — Supporting Information [file JTSA-41-293-s001.pdf]

Supplementary material for “Extracting conditionally heteroscedastic components using ICA”

Jari Miettinen[1], Markus Matilainen[2], Klaus Nordhausen*[3] and Sara Taskinen[4]

[1]Aalto University, Finland
[2]University of Turku, Finland
[3]Vienna University of Technology, Austria
[4]University of Jyvaskyla, Finland

In this supplementary material we provide proofs of the results in the main text as well as asymptotic variances and covariances for Corollary 1 assuming ARMA-GARCH(1,1) model.

Proof of Theorem 1

Write

$$\begin{aligned}\hat{\boldsymbol{T}}(\hat{\boldsymbol{\gamma}}) = b\sum_{\tau\in\mathcal{T}_1}\Bigg(\frac{1}{n}\sum_{t=1}^{n-\tau}\left(\hat{\boldsymbol{\gamma}}^T\boldsymbol{x}_t\hat{\boldsymbol{\gamma}}^T\boldsymbol{x}_{t+\tau}\right)\frac{1}{n}\sum_{t=1}^{n-\tau}\left((\hat{\boldsymbol{\gamma}}^T\boldsymbol{x}_{t+\tau})\boldsymbol{x}_t+(\hat{\boldsymbol{\gamma}}^T\boldsymbol{x}_t)\boldsymbol{x}_{t+\tau}\right)\Bigg)\\ +2(1-b)\sum_{\tau\in\mathcal{T}_2}\Bigg(\frac{1}{n}\sum_{t=1}^{n-\tau}\left((\hat{\boldsymbol{\gamma}}^T\boldsymbol{x}_t)^2(\hat{\boldsymbol{\gamma}}^T\boldsymbol{x}_{t+\tau})^2-1\right)\times\\ \frac{1}{n}\sum_{t=1}^{n-\tau}\left((\hat{\boldsymbol{\gamma}}^T\boldsymbol{x}_t)(\hat{\boldsymbol{\gamma}}^T\boldsymbol{x}_{t+\tau})^2\boldsymbol{x}_t+(\hat{\boldsymbol{\gamma}}^T\boldsymbol{x}_t)^2(\hat{\boldsymbol{\gamma}}^T\boldsymbol{x}_{t+\tau})\boldsymbol{x}_{t+\tau}\right)\Bigg)\quad\text{and}\\ \hat{\boldsymbol{T}}_j := \hat{\boldsymbol{T}}(\hat{\boldsymbol{\gamma}}_j)\end{aligned}$$

*Address for correspondence: Klaus Nordhausen, Institute of Statistics & Mathematical Methods in Economics, Vienna University of Technology, Austria. E-mail: klaus.nordhausen@tuwien.ac.at

The estimating equations of the gSOBI estimate are then

$$\hat{\boldsymbol{\gamma}}_l^T \hat{\boldsymbol{T}}_j = \hat{\boldsymbol{\gamma}}_j^T \hat{\boldsymbol{T}}_l \quad \text{and} \quad \hat{\boldsymbol{\gamma}}_j^T S \hat{\boldsymbol{\gamma}}_l = \delta_{jl}.$$

We begin with

$$\begin{aligned}\sqrt{n}\,\hat{\boldsymbol{\gamma}}_l^T \hat{\boldsymbol{T}}_j =& \sqrt{n}\,(\hat{\boldsymbol{\gamma}}_l - \boldsymbol{e}_l)^T \hat{\boldsymbol{T}}_j \\ &+ \sqrt{n}\,\boldsymbol{e}_l^T \left(\hat{\boldsymbol{T}}_j - \left(2b \sum_{\tau \in \mathcal{T}_1} \mu_{\tau j}^2 - 4(1-b) \sum_{\tau \in \mathcal{T}_2} \nu_{\tau j}(\nu_{\tau j}+1) \right) \boldsymbol{e}_j \right) + o_P(1).\end{aligned}$$

Taylor's and Slutsky's theorems give

$$\begin{aligned}&\sqrt{n}\,\left(\hat{\boldsymbol{T}}_j - \left(2b \sum_{\tau \in \mathcal{T}_1} \mu_{\tau j}^2 - 4(1-w) \sum_{\tau \in \mathcal{T}_2} \nu_{\tau j}(\nu_{\tau j}+1) \right) \boldsymbol{e}_j \right) \\ &= \sqrt{n}\,\left(\boldsymbol{T}_j - \left(2b \sum_{\tau \in \mathcal{T}_1} \mu_{\tau j}^2 - 4(1-b) \sum_{\tau \in \mathcal{T}_2} \nu_{\tau j}(\nu_{\tau j}+1) \right) \boldsymbol{e}_j \right) \\ &\quad + \left(2b \sum_{\tau \in \mathcal{T}_1} \left(\mu_{\tau j}^2 \boldsymbol{e}_j \boldsymbol{e}_j^T + \mu_{\tau j} \mathrm{E}\left(\boldsymbol{x}_t \boldsymbol{x}_{t+\tau} + \boldsymbol{x}_{t+\tau} \boldsymbol{x}_t \right) \right) + 16 \sum_{\tau \in \mathcal{T}_2} \left((\nu_{kj}+1)^2 \boldsymbol{e}_j \boldsymbol{e}_j^T \right.\right. \\ &\quad + \nu_{\tau j} \mathrm{E}\left(2x_{t+k,j}^2 \boldsymbol{x}_t \boldsymbol{x}_t^T + 4x_{t,j} x_{t+k,j} \boldsymbol{x}_t \boldsymbol{x}_{t+k}^T + 4x_{t,j} x_{t+\tau,j} \boldsymbol{x}_{t+\tau} \boldsymbol{x}_t^T \right. \\ &\quad \left.\left.\left. + 2x_{t,j}^2 \boldsymbol{x}_{t+\tau} \boldsymbol{x}_{t+\tau}^T \right)\right)\right) \sqrt{n}\,(\hat{\boldsymbol{\gamma}}_j - \boldsymbol{e}_j) + o_P(1).\end{aligned}$$

After some calculations we obtain

$$\begin{aligned}\sqrt{n}\,\hat{\boldsymbol{\gamma}}_l^T \hat{\boldsymbol{T}}_j =& \left(2b \sum_{\tau \in \mathcal{T}_1} \mu_{\tau j} + 4(1-b) \sum_{\tau \in \mathcal{T}_2} \nu_{kj}(\nu_{\tau j}+1) \right) \sqrt{n}\,\hat{\gamma}_{lj} + \boldsymbol{e}_l^T \sqrt{n}\,\boldsymbol{T}_j \\ &+ \left(2b \sum_{\tau \in \mathcal{T}_1} \mu_{\tau j}\mu_{\tau l} + 4 \sum_{\tau \in \mathcal{T}_2} \nu_{\tau j}\left(1 + 2\mu_{\tau j}\mu_{\tau l} \right) \right) \sqrt{n}\,\hat{\gamma}_{jl} + o_P(1).\end{aligned}$$

Finally, the estimating equations yield the equation

$$\begin{aligned}
&\left(2b\sum_{\tau\in\mathcal{T}_1}\mu_{\tau j}^2+4(1-b)\sum_{\tau\in\mathcal{T}_2}\nu_{\tau j}(\nu_{\tau j}+1)\right)\sqrt{n}\,\left(-\hat{\gamma}_{jl}-\hat{S}_{jl}\right)+\boldsymbol{e}_l^T\sqrt{n}\,\boldsymbol{T}_j\\
&\quad+\left(2b\sum_{\tau\in\mathcal{T}_1}\mu_{\tau j}\mu_{\tau l}+4(1-b)\sum_{\tau\in\mathcal{T}_2}\nu_{\tau j}\left(1+2\mu_{\tau j}\mu_{\tau l}\right)\right)\sqrt{n}\,\hat{\gamma}_{jl}+o_P(1)\\
&=\left(2b\sum_{\tau\in\mathcal{T}_1}\mu_{\tau l}^2+4(1-b)\sum_{\tau\in\mathcal{T}_2}\nu_{\tau l}(\nu_{\tau l}+1)\right)\sqrt{n}\,\hat{\gamma}_{jl}+\boldsymbol{e}_j^T\sqrt{n}\,\boldsymbol{T}_l\\
&\quad+\left(2b\sum_{\tau\in\mathcal{T}_1}\mu_{\tau j}\mu_{\tau l}+4(1-b)\sum_{\tau\in\mathcal{T}_2}\nu_{\tau l}\left(1+2\mu_{\tau j}\mu_{\tau l}\right)\right)\sqrt{n}\,(-\hat{\gamma}_{jl}-\hat{S}_{jl}),
\end{aligned}$$

and hence

$$\begin{aligned}
\sqrt{n}\,\hat{\gamma}_{jl}=&\left(\boldsymbol{e}_l^T\sqrt{n}\,\boldsymbol{T}_j-\boldsymbol{e}_j^T\sqrt{n}\,\boldsymbol{T}_l+\left(2b\sum_{\tau\in\mathcal{T}_1}\mu_{\tau j}(\mu_{\tau l}-\mu_{\tau j})\right.\right.\\
&\left.\left.+4(1-b)\sum_{\tau\in\mathcal{T}_2}\left(\nu_{\tau l}-\nu_{\tau j}(\nu_{\tau j}+1)+2\nu_{\tau l}\mu_{\tau j}\mu_{\tau l}\right)\right)\sqrt{n}\,\hat{S}_{jl}\right)\\
&\times\left(2b\sum_{\tau\in\mathcal{T}_1}\left(\mu_{\tau j}-\mu_{\tau l}\right)^2+4(1-b)\sum_{\tau\in\mathcal{T}_2}\left(\nu_{\tau j}^2+\nu_{\tau l}^2-2(\nu_{\tau j}+\nu_{\tau l})\mu_{\tau j}\mu_{\tau_l}\right)\right)^{-1}.
\end{aligned}$$

Asymptotic variances and covariances for Corollary 1

Assuming an ARMA-GARCH$(1,1)$ model with finite eighth moments, we have the following asymptotic variances. The required expected values of the process $\boldsymbol{x}$ are given below.

$$ASV(\hat{S}_{jl}) = 1 + 2\sum_{k=1}^{K}\left(\sum_{i=0}^{\infty}\psi_{ij}\psi_{i+k,j}\sum_{i=0}^{\infty}\psi_{il}\psi_{i+k,l}\right)$$

$$ASV(\hat{S}_{jj}) = \mathrm{E}\left[x_{tj}^4\right] + 2\sum_{k=1}^{\infty}\mathrm{E}\left[x_{tj}^2 x_{t+k,j}^2\right] - 2$$

$$ASV(\boldsymbol{e}_l^T\boldsymbol{T}_j^v) = 4\sum_{k\in\mathcal{T}_2}\sum_{i\in\mathcal{T}_2}\nu_{kj}\nu_{ij}\sum_{r=-\infty}^{\infty}\left(\mathrm{E}\left[x_{tj}x_{t+k,j}^2 x_{t+r,j}x_{t+r+i,j}^2\right]\mathrm{E}\left[x_{tl}x_{t+r,l}\right]\right.$$
$$+ \mathrm{E}\left[x_{tj}^2 x_{t+k,j}x_{t+r,j}x_{t+r+i,j}^2\right]\mathrm{E}\left[x_{t+k,l}x_{t+r,l}\right] + \mathrm{E}\left[x_{tj}x_{t+k,j}^2 x_{t+r,j}^2 x_{t+r+i,j}\right]$$
$$\left.\times\mathrm{E}\left[x_{tl}x_{t+r+i,l}\right] + \mathrm{E}\left[x_{tj}^2 x_{t+k,j}x_{t+r,j}^2 x_{t+r+i,j}\right]\mathrm{E}\left[x_{t+k,l}x_{t+r+i,l}\right]\right)$$

$$ASV(\boldsymbol{e}_l^T\boldsymbol{T}_j^s) = \sum_{k\in\mathcal{T}_1}\sum_{i\in\mathcal{T}_1}\mu_{kj}\mu_{ij}\sum_{r=-\infty}^{\infty}\left(\mu_{r+i-k,j}\mu_{rl} + \mu_{r-k,j}\mu_{r+i,l}\right.$$
$$\left.+\mu_{r+i,j}\mu_{r-k,l} + \mu_{rj}\mu_{r+i-k,l}\right)$$

$$ASCOV\left(\boldsymbol{e}_l^T\boldsymbol{T}_j^v, \hat{S}_{jl}\right) = 2\sum_{k\in\mathcal{T}_2}\nu_{kj}\sum_{r=-\infty}^{\infty}\left(\mathrm{E}\left[x_{tj}x_{t+k,j}^2 x_{t+r,j}\right]\mu_{rl}\right.$$
$$\left.+\mathrm{E}\left[x_{tj}^2 x_{t+k,j}x_{t+r,j}\right]\mu_{r-k,l}\right)$$

$$ASCOV\left(\boldsymbol{e}_l^T\boldsymbol{T}_j^s, \hat{S}_{jl}\right) = \sum_{k\in\mathcal{T}_2}\mu_{kj}\sum_{r=-\infty}^{\infty}\left(\mu_{r-k,j}\mu_{rl} + \mu_{rj}\mu_{r-k,l}\right)$$

$$ASCOV\left(\boldsymbol{e}_l^T\boldsymbol{T}_j^v, \boldsymbol{e}_j^T\boldsymbol{T}_l^v\right) = 4\sum_{k\in\mathcal{T}_2}\sum_{i\in\mathcal{T}_2}\nu_{kj}\nu_{il}\sum_{r=-\infty}^{\infty}\left(\mathrm{E}\left[x_{tj}x_{t+k,j}^2 x_{t+r,j}\right]\right.$$
$$\times\mathrm{E}\left[x_{t,l}x_{t+r,l}x_{t+r+i,l}^2\right] + \mathrm{E}\left[x_{tj}^2 x_{t+k,j}x_{t+r,j}\right]\mathrm{E}\left[x_{t+k,l}x_{t+r,l}x_{t+r+i,l}^2\right]$$
$$+ \mathrm{E}\left[x_{tj}x_{t+k,j}^2 x_{t+r+i,j}\right]\mathrm{E}\left[x_{tl}x_{t+r,l}^2 x_{t+r+i,l}\right]$$
$$\left.+\mathrm{E}\left[x_{tj}^2 x_{t+k,j}x_{t+r+i,j}\right]\mathrm{E}\left[x_{t+k,l}x_{t+r,l}^2 x_{t+r+i,l}\right]\right)$$

$$ASCOV\left(\boldsymbol{e}_l^T\boldsymbol{T}_j^s, \boldsymbol{e}_j^T\boldsymbol{T}_l^s\right) = \sum_{k\in\mathcal{T}_1}\sum_{i\in\mathcal{T}_1}\mu_{kj}\mu_{il}\sum_{r=-\infty}^{\infty}\left(\mu_{r-k,j}\mu_{r+i,l} + \mu_{r+i-k,j}\mu_{rl}\right.$$

$$\left.+\mu_{rj}\mu_{r+i-k,l} + \mu_{r+i,j}\mu_{r-k,l}\right),$$

$$ASCOV\left(\boldsymbol{e}_l^T\boldsymbol{T}_j^s, \boldsymbol{e}_l^T\boldsymbol{T}_j^v\right) = 2\sum_{k\in\mathcal{T}_1}\sum_{i\in\mathcal{T}_2}\mu_{kj}\nu_{ij}\sum_{r=-\infty}^{\infty}\left(\mathrm{E}\left[x_{tj}x_{t+r,j}x_{t+r+i,j}^2\right]\mu_{r-k,l}\right.$$

$$+\mathrm{E}\left[x_{tj}x_{t+r,j}^2x_{t+r+i,j}\right]\mu_{r+i-k,l} + \mathrm{E}\left[x_{t+k,j}x_{t+r,j}x_{t+r+i,j}^2\right]\mu_{rl}$$

$$\left.+\mathrm{E}\left[x_{t+k,j}x_{t+r,j}^2x_{t+r+i,j}\right]\mu_{r+i,l}\right),$$

$$ASCOV\left(\boldsymbol{e}_j^T\boldsymbol{T}_l^s, \boldsymbol{e}_l^T\boldsymbol{T}_j^v\right) = 2\sum_{k\in\mathcal{T}_1}\sum_{i\in\mathcal{T}_2}\mu_{kl}\nu_{ij}\sum_{r=-\infty}^{\infty}\left(\mathrm{E}\left[x_{t+k,j}x_{t+r,j}x_{t+r+i,j}^2\right]\mu_{rl}\right.$$

$$+\mathrm{E}\left[x_{t+k,j}x_{t+r,j}^2x_{t+r+i,j}\right]\mu_{r+i,l} + \mathrm{E}\left[x_{tj}x_{t+r,j}x_{t+r+i,j}^2\right]\mu_{r-k,l}$$

$$\left.+\mathrm{E}\left[x_{tj}x_{t+r,j}^2x_{t+r+i,j}\right]\mu_{r+i-k,l}\right).$$

Expected values of an ARMA-GARCH$(1,1)$ process

Assume that x and z are from the ARMA-GARCH(1,1) model (1). Then $\mathrm{E}[x_t] = \mathrm{E}[z_t] = 0$ and $\mathrm{E}[x_t^2] = \mathrm{E}[z_t^2] = 1$, and

$$
\begin{aligned}
\mathrm{E}\left[z_t^4\right] &= \frac{3\left(\omega^2 + 2\omega\left(\alpha+\beta\right)\right)}{1-3\alpha^2-2\alpha\beta-\beta^2},\\
\mathrm{E}\left[z_t^6\right] &= \frac{15\left(\omega^2\left(\omega+3\alpha+3\beta\right)+\omega\mathrm{E}\left[z_t^4\right]\left(3\alpha^2+2\alpha\beta+\beta^2\right)\right)}{1-15\alpha^3-9\alpha^2\beta-3\alpha\beta^2-\beta^3},\\
\mathrm{E}\left[z_t^2 z_{t+\tau}^2\right] &= \omega\sum_{k=0}^{\tau-1}\left(\alpha+\beta\right)^k + \left(\alpha+\beta\right)^{\tau-1}\left(\alpha+\beta/3\right)\mathrm{E}\left[z_t^4\right], \quad \tau>0,\\
\mathrm{E}\left[z_t^4 z_{t+\tau}^2\right] &= \omega\sum_{k=0}^{\tau-1}\left(\alpha+\beta\right)^k\mathrm{E}\left[z_t^4\right] + \left(\alpha+\beta\right)^{\tau-1}\left(\alpha+\beta/5\right)\mathrm{E}\left[z_t^6\right], \quad \tau>0,\\
\mathrm{E}\left[z_t^2 z_{t+1}^4\right] &= 3\left(\omega^2+2\omega\left(\alpha+\beta/3\right)+\left(\alpha^2+2\alpha\beta/5+\beta^2/15\right)\mathrm{E}\left[z_t^6\right]\right),\\
\mathrm{E}\left[z_t^2 z_{t+\tau}^4\right] &= \omega^2\sum_{k=0}^{\tau-1}3^{k+1}\left(\alpha^2+2\alpha\beta/3+\beta^2/3\right)^k + 2\omega\left(\alpha+\beta\right)\sum_{k=0}^{\tau-2}3^{k+1}\times\\
&\quad \left(\alpha^2+2\alpha\beta/3+\beta^2/3\right)^k\mathrm{E}\left[z_t^2 z_{t+\tau-k-1}^2\right] + 2\omega\left(\alpha+\beta/3\right)\mathrm{E}\left[z_t^4\right]\\
&\quad + 3^\tau\left(\alpha^2+2\alpha\beta/3+\beta^2/3\right)^{\tau-1}\left(\alpha^2+2\alpha\beta/5+\beta^2/15\right)\mathrm{E}\left[z_t^6\right],\\
&\quad \tau>1,
\end{aligned}
$$

$$
\begin{aligned}
\mathrm{E}\left[z_t^2 z_{t+\tau_1}^2 z_{t+\tau_2}^2\right] &= \omega^2\left(\sum_{i=0}^{\tau_2-\tau_1-1}\sum_{j=0}^{\tau_1-1}c_{i+j,\alpha,\beta} + \sum_{i=1}^{\tau_1}\sum_{j=i}^{\tau_1}\left(1+\delta_{\{j>i\}}c_{\tau_2-\tau_1-1+j-i,\alpha,\beta}h_{i,\alpha,\beta}\right)\right)\\
&\quad + \omega\mathrm{E}\left[z_t^4\right]\left(\alpha+\frac{\beta}{3}\right)\left(\sum_{i=1}^{\tau_1}2c_{\tau_2-1-i}h_{i,\alpha,\beta} + \sum_{i=1}^{\tau_2-\tau_1}2c_{\tau_1-2+i}\right)+\\
&\quad + \mathrm{E}\left[z_t^6\right]\left(\alpha^2+\frac{2\alpha\beta}{5}+\frac{\beta^2}{15}\right)c_{\tau_2-\tau_1-1,\alpha,\beta}h_{\tau_1,\alpha,\beta},
\end{aligned}
$$

where δ is the Kronecker delta and

$$
c_{\tau,\alpha,\beta} = \sum_{k=0}^{\tau}\alpha^k\beta^{\tau-k}\binom{\tau}{k}, \quad h_{\tau,\alpha,\beta} = \sum_{i=0}^{\tau-1}\sum_{k=0}^{\tau}\sum_{r=0}^{\tau}3^r\binom{\tau-1}{i}\binom{i+1}{r}\binom{\tau-i-1}{k-r}.
$$

$$\mathrm{E}\left[x_t^6\right] = \sum_{j=0}^{\infty} \psi_j^6 \mathrm{E}\left[z_t^6\right] + \sum_{j=0}^{\infty} \sum_{k \neq j} 15 \psi_j^4 \psi_k^2 \mathrm{E}\left[z_t^4 z_{t+j-k}^2\right]$$

$$+ \sum_{j=0}^{\infty} \sum_{k \neq j} \sum_{l \neq j, l \neq k} 15 \psi_j^2 \psi_k^2 \psi_l^2 \mathrm{E}\left[z_t^2 z_{t+j-k}^2 z_{t+j-l}^2\right]$$

$$\mathrm{E}\left[x_t^4\right] = \sum_{j=0}^{\infty} \psi_j^4 \mathrm{E}\left[z_t^4\right] + \sum_{j=0}^{\infty} \sum_{k \neq j} 3 \psi_j^2 \psi_k^2 \mathrm{E}\left[z_t^2 z_{t+j-k}^2\right]$$

$$\mathrm{E}\left[x_t^4 x_{t+\tau}^2\right] = \sum_{j=0}^{\infty} \psi_j^4 \psi_{j+\tau}^2 \mathrm{E}\left[z_t^6\right] + \sum_{j=0}^{\infty} \sum_{k \neq j+\tau} \psi_j^4 \psi_k^2 \mathrm{E}\left[z_t^4 z_{t+j-k+\tau}^2\right]$$

$$+ \sum_{j=0}^{\infty} \sum_{k \neq j} \left(6 \psi_j^2 \psi_{j+\tau}^2 \psi_k^2 + 8 \psi_j^3 \psi_{j+\tau} \psi_k \psi_{k+\tau}\right) \mathrm{E}\left[z_t^4 z_{t+j-k}^2\right]$$

$$+ \sum_{j=0}^{\infty} \sum_{k \neq j} \sum_{l \neq j+\tau, l \neq k+\tau} 3 \psi_j^2 \psi_k^2 \psi_l^2 \mathrm{E}\left[z_t^2 z_{t+j-k}^2 z_{t+j-l+\tau}^2\right]$$

$$+ \sum_{j=0}^{\infty} \sum_{k \neq j} \sum_{l \neq j, l \neq k} 12 \psi_j^2 \psi_k \psi_{k+\tau} \psi_l \psi_{l+\tau} \mathrm{E}\left[z_t^2 z_{t+j-k}^2 z_{t+j-l}^2\right]$$

$$\mathrm{E}\left[x_t^4 x_{t+\tau_1} x_{t+\tau_2}\right] = \sum_{j=0}^{\infty} \psi_j^4 \psi_{j+\tau_1} \psi_{j+\tau_2} \mathrm{E}\left[z_t^6\right] + \sum_{j=0}^{\infty} \sum_{k \neq j+\tau_1} \psi_j^4 \psi_k \psi_{k+\tau_2-\tau_1}$$

$$\times \mathrm{E}\left[z_t^4 z_{t+j-k+\tau_1}^2\right] + \sum_{j=0}^{\infty} \sum_{k \neq j} \left(4 \psi_j^3 \psi_{j+\tau_1} \psi_k \psi_{k+\tau_2} + 4 \psi_j^3 \psi_{j+\tau_2} \psi_k \psi_{k+\tau_1}\right.$$

$$\left. + 6 \psi_j^2 \psi_{j+\tau_1} \psi_{j+\tau_2} \psi_k^2\right) \mathrm{E}\left[z_t^4 z_{t+j-k}^2\right] + \sum_{j=0}^{\infty} \sum_{k \neq j} \sum_{l \neq j+\tau_1, l \neq k+\tau_1} 3 \psi_j^2 \psi_k^2 \psi_l \psi_{l+\tau_2-\tau_1}$$

$$\times \mathrm{E}\left[z_t^2 z_{t+j-k}^2 z_{t+j-l+\tau_1}^2\right] + \sum_{j=0}^{\infty} \sum_{k \neq j} \sum_{l \neq j, l \neq k} 12 \psi_j^2 \psi_k \psi_{k+\tau_1} \psi_l \psi_{l+\tau_2}$$

$$\times \mathrm{E}\left[z_t^2 z_{t+j-k}^2 z_{t+j-l}^2\right]$$

$$
\begin{aligned}
\mathrm{E}\left[x_t^3 x_{t+\tau}^3\right] &= \sum_{j=0}^{\infty} \psi_j^3 \psi_{j+\tau}^3 \mathrm{E}\left[z_t^6\right] + \sum_{j=0}^{\infty} \sum_{k \neq j+\tau} 3\psi_j^3 \psi_{j+\tau} \psi_k^2 \mathrm{E}\left[z_t^4 z_{t+j-k+\tau}^2\right] \\
&+ \sum_{j=0}^{\infty} \sum_{k \neq j} \left(9\psi_j^2 \psi_{j+\tau}^2 \psi_k \psi_{k+\tau} + 3\psi_j \psi_{j+\tau}^3 \psi_k^2\right) \mathrm{E}\left[z_t^4 z_{t+j-k}^2\right] \\
&+ \sum_{j=0}^{\infty} \sum_{k \neq j} \sum_{l \neq j+\tau, l \neq k+\tau} 9\psi_j^2 \psi_k \psi_{k+\tau} \psi_l^2 \mathrm{E}\left[z_t^2 z_{t+j-k}^2 z_{t+j-l+\tau}^2\right] \\
&+ \sum_{j=0}^{\infty} \sum_{k \neq j} \sum_{l \neq j, l \neq k} 6\psi_j \psi_{j+\tau} \psi_k \psi_{k+\tau} \psi_l \psi_{l+\tau} \mathrm{E}\left[z_t^2 z_{t+j-k}^2 z_{t+j-l}^2\right]
\end{aligned}
$$

$$
\begin{aligned}
\mathrm{E}\left[x_t^3 x_{t+\tau_1}^2 x_{t+\tau_2}\right] &= \sum_{j=0}^{\infty} \psi_j^3 \psi_{j+\tau_1}^2 \psi_{j+\tau_2} \mathrm{E}\left[z_t^6\right] + \sum_{j=0}^{\infty} \sum_{k \neq j+\tau_1} \left(\psi_j^3 \psi_{j+\tau_2} \psi_k^2\right. \\
&\left.+ 2\psi_j^3 \psi_{j+\tau_1} \psi_k \psi_{k+\tau_2-\tau_1}\right) \mathrm{E}\left[z_t^4 z_{t+j-k+\tau_1}^2\right] + \sum_{j=0}^{\infty} \sum_{k \neq j} \left(3\psi_j^2 \psi_{j+\tau_1}^2 \psi_k \psi_{k+\tau_2}\right. \\
&\left.+ 6\psi_j^2 \psi_{j+\tau_1} \psi_{j+\tau_2} \psi_k \psi_{k+\tau_1} + 3\psi_j \psi_{j+\tau_1}^2 \psi_{j+\tau_2} \psi_k^2\right) \mathrm{E}\left[z_t^4 z_{t+j-k}^2\right] \\
&+ \sum_{j=0}^{\infty} \sum_{k \neq j} \sum_{l \neq j+\tau_1, l \neq k+\tau_1} \left(6\psi_j^2 \psi_k \psi_{k+\tau_1} \psi_l \psi_{l+\tau_2-\tau_1} + 3\psi_j^2 \psi_k \psi_{k+\tau_2} \psi_l^2\right) \\
&\times \mathrm{E}\left[z_t^2 z_{t+j-k}^2 z_{t+j-l+\tau_1}^2\right] + \sum_{j=0}^{\infty} \sum_{k \neq j} \sum_{l \neq j, l \neq k} 6\psi_j \psi_{j+\tau_1} \psi_k \psi_{k+\tau_1} \psi_l \psi_{l+\tau_2} \\
&\times \mathrm{E}\left[z_t^2 z_{t+j-k}^2 z_{t+j-l}^2\right]
\end{aligned}
$$

$$
\mathrm{E}\left[x_t^3 x_{t+\tau}\right] = \sum_{j=0}^{\infty} \psi_j^3 \psi_{j+\tau} \mathrm{E}\left[z_t^4\right] + \sum_{j=0}^{\infty} \sum_{k \neq j} 3\psi_j^2 \psi_k \psi_{k+\tau} \mathrm{E}\left[z_t^2 z_{t+j-k}^2\right]
$$

$$
\begin{aligned}
\mathrm{E}\left[x_t^3 x_{t+\tau_1} x_{t+\tau_2}^2\right] &= \sum_{j=0}^{\infty} \psi_j^3 \psi_{j+\tau_1} \psi_{j+\tau_2}^2 \mathrm{E}\left[z_t^6\right] + \sum_{j=0}^{\infty} \sum_{k \neq j+\tau_2} \psi_j^3 \psi_{j+\tau_1} \psi_k^2 \\
&\times \mathrm{E}\left[z_t^4 z_{t+j-k+\tau_2}^2\right] + \sum_{j=0}^{\infty} \sum_{k \neq j+\tau_1} 2\psi_j^3 \psi_{j+\tau_2} \psi_k \psi_{k+\tau_2-\tau_1} \mathrm{E}\left[z_t^4 z_{t+j-k+\tau_1}^2\right] \\
&+ \sum_{j=0}^{\infty} \sum_{k \neq j} \left(3\psi_j^2 \psi_{j+\tau_2}^2 \psi_k \psi_{k+\tau_1} + 6\psi_j^2 \psi_{j+\tau_1} \psi_{j+\tau_2} \psi_k \psi_{k+\tau_2}\right. \\
&\left. +3\psi_j \psi_{j+\tau_1} \psi_{j+\tau_2}^2 \psi_k^2\right) \mathrm{E}\left[z_t^4 z_{t+j-k}^2\right] \\
&+ \sum_{j=0}^{\infty} \sum_{k \neq j} \sum_{l \neq j+\tau_2, l \neq k+\tau_2} 3\psi_j^2 \psi_k \psi_{k+\tau_1} \psi_l^2 \mathrm{E}\left[z_t^2 z_{t+j-k}^2 z_{t+j-l+\tau_2}^2\right] \\
&+ \sum_{j=0}^{\infty} \sum_{k \neq j} \sum_{l \neq j+\tau_1, l \neq k+\tau_1} 6\psi_j^2 \psi_k \psi_{k+\tau_2} \psi_l \psi_{l+\tau_2-\tau_1} \mathrm{E}\left[z_t^2 z_{t+j-k}^2 z_{t+j-l+\tau_1}^2\right] \\
&+ \sum_{j=0}^{\infty} \sum_{k \neq j} \sum_{l \neq j, l \neq k} 6\psi_j \psi_{j+\tau_1} \psi_k \psi_{k+\tau_2} \psi_l \psi_{l+\tau_2} \mathrm{E}\left[z_t^2 z_{t+j-k}^2 z_{t+j-l}^2\right]
\end{aligned}
$$

$$
\begin{aligned}
\mathrm{E}\left[x_t^2 x_{t+\tau}^4\right] &= \sum_{j=0}^{\infty} \psi_j^2 \psi_{j+\tau}^4 \mathrm{E}\left[z_t^6\right] + \sum_{j=0}^{\infty} \sum_{k \neq j+\tau} \left(6\psi_j^2 \psi_{j+\tau}^2 \psi_k^2 \mathrm{E}\left[z_t^4 z_{t+j-k+\tau}^2\right]\right. \\
&\left. +\psi_j^2 \psi_k^4 \mathrm{E}\left[z_t^2 z_{t+j-k+\tau}^4\right]\right) + \sum_{j=0}^{\infty} \sum_{k \neq j} 8\psi_j \psi_{j+\tau}^3 \psi_k \psi_{k+\tau} \mathrm{E}\left[z_t^4 z_{t+j-k}^2\right] \\
&+ \sum_{j=0}^{\infty} \sum_{k \neq j+\tau} \sum_{l \neq j+\tau, l \neq k} 3\psi_j^2 \psi_k^2 \psi_l^2 \mathrm{E}\left[z_t^2 z_{t+j-k+\tau}^2 z_{t+j-l+\tau}^2\right] \\
&+ \sum_{j=0}^{\infty} \sum_{k \neq j} \sum_{l \neq j, l \neq k} 12\psi_j \psi_{j+\tau} \psi_k \psi_{k+\tau} \psi_l^2 \mathrm{E}\left[z_t^2 z_{t+j-k}^2 z_{t+j-l}^2\right]
\end{aligned}
$$

$$\mathrm{E}\left[x_t^2 x_{t+\tau_1}^3 x_{t+\tau_2}\right] = \sum_{j=0}^{\infty} \psi_j^2 \psi_{j+\tau_1}^3 \psi_{j+\tau_2} \mathrm{E}\left[z_t^6\right] + \sum_{j=0}^{\infty} \sum_{k \neq j+\tau_1} \left(\left(3\psi_j^2 \psi_{j+\tau_1}^2 \psi_k \psi_{k+\tau_2-\tau_1} \right.\right.$$

$$\left. + 3\psi_j^2 \psi_{j+\tau_1} \psi_{j+\tau_2} \psi_k^2 \right) \mathrm{E}\left[z_t^4 z_{t+j-k+\tau_1}^2\right] + \psi_j^2 \psi_k^3 \psi_{k+\tau_2-\tau_1} \mathrm{E}\left[z_t^2 z_{t+j-k+\tau_1}^4\right] \Big)$$

$$+ \sum_{j=0}^{\infty} \sum_{k \neq j} \left(2\psi_j \psi_{j+\tau_1}^3 \psi_k \psi_{k+\tau_2} + 6\psi_j \psi_{j+\tau_1}^2 \psi_{j+\tau_2} \psi_k \psi_{k+\tau_1} \right) \mathrm{E}\left[z_t^4 z_{t+j-k}^2\right]$$

$$+ \sum_{j=0}^{\infty} \sum_{k \neq j+\tau_1} \sum_{l \neq j+\tau_1, l \neq k} 3\psi_j^2 \psi_k^2 \psi_l \psi_{l+\tau_2-\tau_1} \mathrm{E}\left[z_t^2 z_{t+j-k+\tau_1}^2 z_{t+j-l+\tau_1}^2\right]$$

$$+ \sum_{j=0}^{\infty} \sum_{k \neq j} \sum_{l \neq j+\tau_1, l \neq k+\tau_1} \left(6\psi_j \psi_{j+\tau_1} \psi_k \psi_{k+\tau_1} \psi_l \psi_{l+\tau_2-\tau_1} + 6\psi_j \psi_{j+\tau_1} \psi_k \psi_{k+\tau_2} \psi_l^2 \right)$$

$$\times \mathrm{E}\left[z_t^2 z_{t+j-k}^2 z_{t+j-l+\tau_1}^2\right]$$

$$\mathrm{E}\left[x_t^2 x_{t+\tau}^2\right] = \sum_{j=0}^{\infty} \psi_j^2 \psi_{j+\tau}^2 \mathrm{E}\left[z_t^4\right] + \sum_{j=0}^{\infty} \sum_{k \neq j} 2\psi_j \psi_{j+\tau} \psi_k \psi_{k+\tau} \mathrm{E}\left[z_t^2 z_{t+j-k}^2\right]$$

$$+ \sum_{j=0}^{\infty} \sum_{k \neq j+\tau} \psi_j^2 \psi_k^2 \mathrm{E}\left[z_t^2 z_{t+j-k+\tau}^2\right]$$

$$\mathrm{E}\left[x_t^2 x_{t+\tau_1}^2 x_{t+\tau_2}^2\right] = \sum_{j=0}^{\infty} \psi_j^2 \psi_{j+\tau_1}^2 \psi_{j+\tau_2}^2 \mathrm{E}\left[z_t^6\right] + \sum_{j=0}^{\infty} \sum_{k \neq j+\tau_2} \psi_j^2 \psi_{j+\tau_1}^2 \psi_k^2$$

$$\times \mathrm{E}\left[z_t^4 z_{t+j-k+\tau_2}^2\right] + \sum_{j=0}^{\infty} \sum_{k \neq j+\tau_1} \left(\left(\psi_j^2 \psi_{j+\tau_2}^2 \psi_k^2 + 4\psi_j^2 \psi_{j+\tau_1} \psi_{j+\tau_2} \psi_k \psi_{k+\tau_2-\tau_1} \right) \right.$$

$$\left. \times \mathrm{E}\left[z_t^4 z_{t+j-k+\tau_1}^2\right] + \psi_j^2 \psi_k^2 \psi_{k+\tau_2-\tau_1}^2 \mathrm{E}\left[z_t^2 z_{t+j-k+\tau_1}^4\right] \right)$$

$$+ \sum_{j=0}^{\infty} \sum_{k \neq j} \left(4\psi_j \psi_{j+\tau_1}^2 \psi_{j+\tau_2} \psi_k \psi_{k+\tau_2} + 4\psi_j \psi_{j+\tau_1} \psi_{j+\tau_2}^2 \psi_k \psi_{k+\tau_1} \right) \times \mathrm{E}\left[z_t^4 z_{t+j-k}^2\right]$$

$$+ \sum_{j=0}^{\infty} \sum_{k \neq j+\tau_1} \sum_{l \neq j+\tau_2, l \neq k+\tau_2-\tau_1} \psi_j^2 \psi_k^2 \psi_l^2 \mathrm{E}\left[z_t^2 z_{t+j-k+\tau_1}^2 z_{t+j-l+\tau_2}^2\right]$$

$$+ \sum_{j=0}^{\infty} \sum_{k \neq j} \sum_{l \neq j+\tau_2, l \neq k+\tau_2} 2\psi_j \psi_{j+\tau_1} \psi_k \psi_{k+\tau_1} \psi_l^2 \mathrm{E}\left[z_t^2 z_{t+j-k}^2 z_{t+j-l+\tau_2}^2\right]$$

$$+ \sum_{j=0}^{\infty} \sum_{k \neq j+\tau_1} \sum_{l \neq j+\tau_1, l \neq k} 2\psi_j^2 \psi_k \psi_{k+\tau_2-\tau_1} \psi_l \psi_{l+\tau_2-\tau_1} \mathrm{E}\left[z_t^2 z_{t+j-k+\tau_1}^2 z_{t+j-l+\tau_1}^2\right]$$

$$+ \sum_{j=0}^{\infty} \sum_{k \neq j} \sum_{l \neq j+\tau_1, l \neq k+\tau_1} \left(2\psi_j \psi_{j+\tau_2} \psi_k \psi_{k+\tau_2} \psi_l^2 + 8\psi_j \psi_{j+\tau_1} \psi_k \psi_{k+\tau_2} \psi_l \psi_{l+\tau_2-\tau_1} \right)$$

$$\times \mathrm{E}\left[z_t^2 z_{t+j-k+\tau_1}^2 z_{t+j-l+\tau_1}^2\right]$$

$$\mathrm{E}\left[x_t^2 x_{t+\tau_1}^2 x_{t+\tau_2} x_{t+\tau_3}\right] = \sum_{j=0}^{\infty} \psi_j^2 \psi_{j+\tau_1}^2 \psi_{j+\tau_2} \psi_{j+\tau_3} \mathrm{E}\left[z_t^6\right] + \sum_{j=0}^{\infty} \sum_{k \neq j+\tau_2} \psi_j^2 \psi_{j+\tau_1}^2$$

$$\times \psi_k \psi_{k+\tau_3-\tau_2} \mathrm{E}\left[z_t^4 z_{t+j-k+\tau_2}^2\right] + \sum_{j=0}^{\infty} \sum_{k \neq j+\tau_1} \Big(\big(2\psi_j^2 \psi_{j+\tau_1} \psi_{j+\tau_2} \psi_k \psi_{k+\tau_3-\tau_1}$$

$$+2\psi_j^2 \psi_{j+\tau_1} \psi_{j+\tau_3} \psi_k \psi_{k+\tau_2-\tau_1} + \psi_j^2 \psi_{j+\tau_2} \psi_{j+\tau_3} \psi_k^2\big) \mathrm{E}\left[z_t^4 z_{t+j-k+\tau_1}^2\right] + \psi_j^2 \psi_k^2$$

$$\times \psi_{k+\tau_2-\tau_1} \psi_{k+\tau_3-\tau_1} \mathrm{E}\left[z_t^2 z_{t+j-k+\tau_1}^4\right]\Big) + \sum_{j=0}^{\infty} \sum_{k \neq j} \big(2\psi_j \psi_{j+\tau_1}^2 \psi_{j+\tau_2} \psi_k \psi_{k+\tau_3}$$

$$+2\psi_j \psi_{j+\tau_1}^2 \psi_{j+\tau_3} \psi_k \psi_{k+\tau_2} + 4\psi_j \psi_{j+\tau_1} \psi_{j+\tau_2} \psi_{j+\tau_3} \psi_k \psi_{k+\tau_1}\big) \mathrm{E}\left[z_t^4 z_{t+j-k}^2\right]$$

$$+ \sum_{j=0}^{\infty} \sum_{k \neq j+\tau_1} \sum_{l \neq j+\tau_2, l \neq k+\tau_2-\tau_1} \psi_j^2 \psi_k^2 \psi_l \psi_{l+\tau_3-\tau_2} \mathrm{E}\left[z_t^2 z_{t+j-k+\tau_1}^2 z_{t+j-l+\tau_2}^2\right]$$

$$+ \sum_{j=0}^{\infty} \sum_{k \neq j} \sum_{l \neq j+\tau_2, l \neq k+\tau_2} 2\psi_j \psi_{j+\tau_1} \psi_k \psi_{k+\tau_1} \psi_l \psi_{l+\tau_3-\tau_2} \mathrm{E}\left[z_t^2 z_{t+j-k}^2 z_{t+j-l+\tau_2}^2\right]$$

$$+ \sum_{j=0}^{\infty} \sum_{k \neq j+\tau_1} \sum_{l \neq j+\tau_1, l \neq k} 2\psi_j^2 \psi_k \psi_{k+\tau_2-\tau_1} \psi_l \psi_{l+\tau_3-\tau_1} \mathrm{E}\left[z_t^2 z_{t+j-k+\tau_1}^2 z_{t+j-l+\tau_1}^2\right]$$

$$+ \sum_{j=0}^{\infty} \sum_{k \neq j} \sum_{l \neq j+\tau_1, l \neq k+\tau_1} \big(4\psi_j \psi_{j+\tau_1} \psi_k \psi_{k+\tau_2} \psi_l \psi_{l+\tau_3-\tau_1} + 4\psi_j \psi_{j+\tau_1} \psi_k \psi_{k+\tau_3} \psi_l$$

$$\times \psi_{l+\tau_2-\tau_1} + 2\psi_j \psi_{j+\tau_2} \psi_k \psi_{k+\tau_3} \psi_l^2\big) \mathrm{E}\left[z_t^2 z_{t+j-k}^2 z_{t+j-l+\tau_1}^2\right]$$

$$\mathrm{E}\left[x_t^2 x_{t+\tau_1} x_{t+\tau_2}^3\right] = \sum_{j=0}^{\infty} \psi_j^2 \psi_{j+\tau_1} \psi_{j+\tau_2}^3 \mathrm{E}\left[z_t^6\right] + \sum_{j=0}^{\infty} \sum_{k \neq j+\tau_2} 3\psi_j^2 \psi_{j+\tau_1} \psi_{j+\tau_2} \psi_k^2$$

$$\times \mathrm{E}\left[z_t^4 z_{t+j-k+\tau_2}^2\right] + \sum_{j=0}^{\infty} \sum_{k \neq j+\tau_1} \left(3\psi_j^2 \psi_{j+\tau_2}^2 \psi_k \psi_{k+\tau_2-\tau_1} \mathrm{E}\left[z_t^4 z_{t+j-k+\tau_1}^2\right]\right.$$

$$\left.+\psi_j^2 \psi_k \psi_{k+\tau_2-\tau_1}^3 \mathrm{E}\left[z_t^2 z_{t+j-k+\tau_1}^4\right]\right) + \sum_{j=0}^{\infty} \sum_{k \neq j} \left(6\psi_j \psi_{j+\tau_1} \psi_{j+\tau_2}^2 \psi_k \psi_{k+\tau_2}\right.$$

$$\left.+2\psi_j \psi_{j+\tau_2}^3 \psi_k \psi_{k+\tau_1}\right) \mathrm{E}\left[z_t^4 z_{t+j-k}^2\right]$$

$$+ \sum_{j=0}^{\infty} \sum_{k \neq j+\tau_1} \sum_{l \neq j+\tau_2, l \neq k+\tau_2-\tau_1} 3\psi_j^2 \psi_k \psi_{k+\tau_2-\tau_1} \psi_l^2 \mathrm{E}\left[z_t^2 z_{t+j-k+\tau_1}^2 z_{t+j-l+\tau_2}^2\right]$$

$$+ \sum_{j=0}^{\infty} \sum_{k \neq j} \sum_{l \neq j+\tau_2, l \neq k+\tau_2} 6\psi_j \psi_{j+\tau_1} \psi_k \psi_{k+\tau_2} \psi_l^2 \mathrm{E}\left[z_t^2 z_{t+j-k}^2 z_{t+j-l+\tau_2}^2\right]$$

$$+ \sum_{j=0}^{\infty} \sum_{k \neq j} \sum_{l \neq j+\tau_1, l \neq k+\tau_1} 6\psi_j \psi_{j+\tau_2} \psi_k \psi_{k+\tau_2} \psi_l \psi_{l+\tau_2-\tau_1} \mathrm{E}\left[z_t^2 z_{t+j-k}^2 z_{t+j-l+\tau_1}^2\right]$$

$$
\begin{aligned}
\mathrm{E}\left[x_t^2 x_{t+\tau_1} x_{t+\tau_2}^2 x_{t+\tau_3}\right] &= \sum_{j=0}^{\infty} \psi_j^2 \psi_{j+\tau_1} \psi_{j+\tau_2}^2 \psi_{j+\tau_3} \mathrm{E}\left[z_t^6\right] + \sum_{j=0}^{\infty} \sum_{k \neq j+\tau_2} \left(2\psi_j^2 \psi_{j+\tau_1}\right.\\
&\left.\times \psi_{j+\tau_2} \psi_k \psi_{k+\tau_3-\tau_2} + \psi_j^2 \psi_{j+\tau_1} \psi_{j+\tau_3} \psi_k^2\right) \mathrm{E}\left[z_t^4 z_{t+j-k+\tau_2}^2\right] + \sum_{j=0}^{\infty} \sum_{k \neq j+\tau_1} \left(\left(\psi_j^2 \psi_{j+\tau_2}^2\right.\right.\\
&\left.\times \psi_k \psi_{k+\tau_3-\tau_1} + 2\psi_j^2 \psi_{j+\tau_2} \psi_{j+\tau_3} \psi_k \psi_{k+\tau_2-\tau_1}\right) \mathrm{E}\left[z_t^4 z_{t+j-k+\tau_1}^2\right] + \psi_j^2 \psi_k \psi_{k+\tau_2-\tau_1}^2\\
&\left.\times \psi_{k+\tau_3-\tau_1} \mathrm{E}\left[z_t^2 z_{t+j-k+\tau_1}^4\right]\right) + \sum_{j=0}^{\infty} \sum_{k \neq j} \left(2\psi_j \psi_{j+\tau_1} \psi_{j+\tau_2}^2 \psi_k \psi_{k+\tau_3} + 4\psi_j \psi_{j+\tau_1}\right.\\
&\left.\times \psi_{j+\tau_2} \psi_{j+\tau_3} \psi_k \psi_{k+\tau_2} + 2\psi_j \psi_{j+\tau_2}^2 \psi_{j+\tau_3} \psi_k \psi_{k+\tau_1}\right) \mathrm{E}\left[z_t^4 z_{t+j-k}^2\right]\\
&+ \sum_{j=0}^{\infty} \sum_{k \neq j+\tau_1} \sum_{l \neq j+\tau_2, l \neq k+\tau_2-\tau_1} \left(2\psi_j^2 \psi_k \psi_{k+\tau_2-\tau_1} \psi_l \psi_{l+\tau_3-\tau_2} + \psi_j^2 \psi_k \psi_{k+\tau_3-\tau_1} \psi_l^2\right)\\
&\times \mathrm{E}\left[z_t^2 z_{t+j-k+\tau_1}^2 z_{t+j-l+\tau_2}^2\right] + \sum_{j=0}^{\infty} \sum_{k \neq j} \sum_{l \neq j+\tau_2, l \neq k+\tau_2} (4\psi_j \psi_{j+\tau_1} \psi_k \psi_{k+\tau_2} \psi_l \psi_{l+\tau_3-\tau_2}\\
&+2\psi_j \psi_{j+\tau_1} \psi_k \psi_{k+\tau_3} \psi_l^2) \mathrm{E}\left[z_t^2 z_{t+j-k}^2 z_{t+j-l+\tau_2}^2\right] + \sum_{j=0}^{\infty} \sum_{k \neq j} \sum_{l \neq j+\tau_1, l \neq k+\tau_1} (2\psi_j \psi_{j+\tau_2}\\
&\times \psi_k \psi_{k+\tau_2} \psi_l \psi_{l+\tau_3-\tau_1} + 4\psi_j \psi_{j+\tau_2} \psi_k \psi_{k+\tau_3} \psi_l \psi_{l+\tau_2-\tau_1}) \mathrm{E}\left[z_t^2 z_{t+j-k}^2 z_{t+j-l+\tau_1}^2\right]
\end{aligned}
$$

$$
\begin{aligned}
\mathrm{E}\left[x_t^2 x_{t+\tau_1} x_{t+\tau_2}\right] &= \sum_{j=0}^{\infty} \psi_j^2 \psi_{j+\tau_1} \psi_{j+\tau_2} \mathrm{E}\left[z_t^4\right] + \sum_{j=0}^{\infty} \sum_{k \neq j+\tau_1} \psi_j^2 \psi_k \psi_{k+\tau_2-\tau_1}\\
&\times \mathrm{E}\left[z_t^2 z_{t+j-k+\tau_1}^2\right] + \sum_{j=0}^{\infty} \sum_{k \neq j} 2\psi_j \psi_{j+\tau_1} \psi_k \psi_{k+\tau_2} \mathrm{E}\left[z_t^2 z_{t+j-k+\tau}^2\right]
\end{aligned}
$$

$$
\begin{aligned}
&\mathrm{E}\left[x_t^2 x_{t+\tau_1} x_{t+\tau_2} x_{t+\tau_3}^2\right] = \sum_{j=0}^{\infty} \psi_j^2 \psi_{j+\tau_1} \psi_{j+\tau_2} \psi_{j+\tau_3}^2 \mathrm{E}\left[z_t^6\right] + \sum_{j=0}^{\infty} \sum_{k \neq j+\tau_3} \psi_j^2 \psi_{j+\tau_1} \psi_{j+\tau_2} \\
&\times \psi_k^2 \mathrm{E}\left[z_t^4 z_{t+j-k+\tau_3}^2\right] + \sum_{j=0}^{\infty} \sum_{k \neq j+\tau_2} 2\psi_j^2 \psi_{j+\tau_1} \psi_{j+\tau_3} \psi_k \psi_{k+\tau_3-\tau_2} \mathrm{E}\left[z_t^4 z_{t+j-k+\tau_2}^2\right] \\
&+ \sum_{j=0}^{\infty} \sum_{k \neq j+\tau_1} \Big(\big(2\psi_j^2 \psi_{j+\tau_2} \psi_{j+\tau_3} \psi_k \psi_{k+\tau_3-\tau_1} + \psi_j^2 \psi_{j+\tau_3}^2 \psi_k \psi_{k+\tau_2-\tau_1}\big) \mathrm{E}\left[z_t^4 z_{t+j-k+\tau_1}^2\right] \\
&+ \psi_j^2 \psi_k \psi_{k+\tau_2-\tau_1} \psi_{k+\tau_3-\tau_1}^2 \mathrm{E}\left[z_t^2 z_{t+j-k+\tau_1}^4\right]\Big) + \sum_{j=0}^{\infty} \sum_{k \neq j} \big(4\psi_j \psi_{j+\tau_1} \psi_{j+\tau_2} \psi_{j+\tau_3} \psi_k \psi_{k+\tau_3} \\
&+ 2\psi_j \psi_{j+\tau_1} \psi_{j+\tau_3}^2 \psi_k \psi_{k+\tau_2} + 2\psi_j \psi_{j+\tau_2} \psi_{j+\tau_3}^2 \psi_k \psi_{k+\tau_1}\big) \mathrm{E}\left[z_t^4 z_{t+j-k}^2\right] \\
&+ \sum_{j=0}^{\infty} \sum_{k \neq j+\tau_1} \sum_{l \neq j+\tau_3, l \neq k+\tau_3-\tau_1} \psi_j^2 \psi_k \psi_{k+\tau_2-\tau_1} \psi_l^2 \mathrm{E}\left[z_t^2 z_{t+j-k+\tau_1}^2 z_{t+j-l+\tau_3}^2\right] \\
&+ \sum_{j=0}^{\infty} \sum_{k \neq j} \sum_{l \neq j+\tau_3, l \neq k+\tau_3} 2\psi_j \psi_{j+\tau_1} \psi_k \psi_{k+\tau_2} \psi_l^2 \mathrm{E}\left[z_t^2 z_{t+j-k}^2 z_{t+j-l+\tau_3}^2\right] \\
&+ \sum_{j=0}^{\infty} \sum_{k \neq j+\tau_1} \sum_{l \neq j+\tau_2, l \neq k+\tau_2-\tau_1} 2\psi_j^2 \psi_k \psi_{k+\tau_3-\tau_1} \psi_l \psi_{l+\tau_3-\tau_2} \mathrm{E}\left[z_t^2 z_{t+j-k+\tau_1}^2 z_{t+j-l+\tau_2}^2\right] \\
&+ \sum_{j=0}^{\infty} \sum_{k \neq j} \sum_{l \neq j+\tau_2, l \neq k+\tau_2} 4\psi_j \psi_{j+\tau_1} \psi_k \psi_{k+\tau_3} \psi_l \psi_{l+\tau_3-\tau_2} \mathrm{E}\left[z_t^2 z_{t+j-k}^2 z_{t+j-l+\tau_2}^2\right] \\
&+ \sum_{j=0}^{\infty} \sum_{k \neq j} \sum_{l \neq j+\tau_1, l \neq k+\tau_1} \left(4\psi_j \psi_{j+\tau_2} \psi_k \psi_{k+\tau_3} \psi_l \psi_{l+\tau_3-\tau_1} + 2\psi_j \psi_{j+\tau_3} \psi_k \psi_{k+\tau_3} \psi_l \psi_{l+\tau_2-\tau_1}\right) \\
&\times \mathrm{E}\left[z_t^2 z_{t+j-k}^2 z_{t+j-l+\tau_1}^2\right]
\end{aligned}
$$

$$\mathrm{E}\left[x_t x_{t+\tau_1}^4 x_{t+\tau_2}\right] = \sum_{j=0}^{\infty} \psi_j \psi_{j+\tau_1}^4 \psi_{j+\tau_2} \mathrm{E}\left[z_t^6\right] + \sum_{j=0}^{\infty} \sum_{k \neq j+\tau_1} \Big(\big(4\psi_j \psi_{j+\tau_1}^3 \psi_k \psi_{k+\tau_2-\tau_1}$$

$$+6\psi_j \psi_{j+\tau_1}^2 \psi_{j+\tau_2} \psi_k^2 \big) \mathrm{E}\left[z_t^4 z_{t+j-k+\tau_1}^2\right] + \psi_j \psi_{j+\tau_2} \psi_k + 4\psi_j \psi_{j+\tau_2} \psi_k^3 \psi_{k+\tau_2-\tau_1} \mathrm{E}\left[z_t^2 z_{t+j-k+\tau_1}^4\right] \Big)$$

$$+ \sum_{j=0}^{\infty} \sum_{k \neq j+\tau_1} \sum_{l \neq j+\tau_1, l \neq k} \left(12\psi_j \psi_{j+\tau_1} \psi_k \psi_{k+\tau_2-\tau_1} \psi_l^2 + 3\psi_j \psi_{j+\tau_2} \psi_k^2 \psi_l^2 \right) \times$$

$$\mathrm{E}\left[z_t^2 z_{t+j-k+\tau_1}^2 z_{t+j-l+\tau_1}^2\right]$$

$$\mathrm{E}\left[x_t x_{t+\tau}^3\right] = \sum_{j=0}^{\infty} \psi_j \psi_{j+\tau}^3 \mathrm{E}\left[z_t^4\right] + \sum_{j=0}^{\infty} \sum_{k \neq j+\tau} 3\psi_j \psi_{j+\tau} \psi_k^2 \mathrm{E}\left[z_t^2 z_{t+j-k+\tau}^2\right]$$

$$\mathrm{E}\left[x_t x_{t+\tau_1}^3 x_{t+\tau_2}^2\right] = \sum_{j=0}^{\infty} \psi_j \psi_{j+\tau_1}^3 \psi_{j+\tau_2}^2 \mathrm{E}\left[z_t^6\right] + \sum_{j=0}^{\infty} \sum_{k \neq j+\tau_2} \psi_j \psi_{j+\tau_1}^3 \psi_k^2 \mathrm{E}\left[z_t^4 z_{t+j-k+\tau_2}^2\right]$$

$$+ \sum_{j=0}^{\infty} \sum_{k \neq j+\tau_1} \Big(\big(6\psi_j \psi_{j+\tau_1}^2 \psi_{j+\tau_2} \psi_k \psi_{k+\tau_2-\tau_1} + 3\psi_j \psi_{j+\tau_1} \psi_{j+\tau_2}^2 \psi_k^2 \big) \mathrm{E}\left[z_t^4 z_{t+j-k+\tau_1}^2\right]$$

$$+ \left(3\psi_j \psi_{j+\tau_1} \psi_k^2 \psi_{k+\tau_2-\tau_1}^2 + 2\psi_j \psi_{j+\tau_2} \psi_k^3 \psi_{k+\tau_2-\tau_1} \right) \mathrm{E}\left[z_t^2 z_{t+j-k+\tau_1}^4\right] \Big)$$

$$+ \sum_{j=0}^{\infty} \sum_{k \neq j+\tau_1} \sum_{l \neq j+\tau_2, l \neq k+\tau_2-\tau_1} 3\psi_j \psi_{j+\tau_1} \psi_k^2 \psi_l^2 \mathrm{E}\left[z_t^2 z_{t+j-k+\tau_1}^2 z_{t+j-l+\tau_2}^2\right]$$

$$+ \sum_{j=0}^{\infty} \sum_{k \neq j+\tau_1} \sum_{l \neq j+\tau_1, l \neq k} \left(6\psi_j \psi_{j+\tau_1} \psi_k \psi_{k+\tau_2-\tau_1} \psi_l \psi_{l+\tau_2-\tau_1} + 6\psi_j \psi_{j+\tau_2} \psi_k^2 \psi_l \psi_{l+\tau_2-\tau_1} \right)$$

$$\times \mathrm{E}\left[z_t^2 z_{t+j-k}^2 z_{t+j-l+\tau_1}^2\right]$$

$$\mathrm{E}\left[x_t x_{t+\tau_1}^2 x_{t+\tau_2}^3\right] = \sum_{j=0}^{\infty} \psi_j \psi_{j+\tau_1}^2 \psi_{j+\tau_2}^3 \mathrm{E}\left[z_t^6\right] + \sum_{j=0}^{\infty} \sum_{k \neq j+\tau_2} 3\psi_j \psi_{j+\tau_1}^2 \psi_{j+\tau_2} \psi_k^2$$

$$\times \mathrm{E}\left[z_t^4 z_{t+j-k+\tau_2}^2\right] + \sum_{j=0}^{\infty} \sum_{k \neq j+\tau_1} \left(\left(6\psi_j \psi_{j+\tau_1} \psi_{j+\tau_2}^2 \psi_k \psi_{k+\tau_2-\tau_1} + \psi_j \psi_{j+\tau_2}^3 \psi_k^2\right)\right.$$

$$\times \mathrm{E}\left[z_t^4 z_{t+j-k+\tau_1}^2\right] + \left(2\psi_j \psi_{j+\tau_1} \psi_k \psi_{k+\tau_2-\tau_1}^3 + 3\psi_j \psi_{j+\tau_2} \psi_k^2 \psi_{k+\tau_2-\tau_1}^2\right)$$

$$\left.\times \mathrm{E}\left[z_t^2 z_{t+j-k+\tau_1}^4\right]\right) + \sum_{j=0}^{\infty} \sum_{k \neq j+\tau_1} \sum_{l \neq j+\tau_2, l \neq k+\tau_2-\tau_1} \left(6\psi_j \psi_{j+\tau_1} \psi_k \psi_{k+\tau_2-\tau_1} \psi_l^2\right.$$

$$\left.+3\psi_j \psi_{j+\tau_2} \psi_k^2 \psi_l^2\right) \mathrm{E}\left[z_t^2 z_{t+j-k+\tau_1}^2 z_{t+j-l+\tau_2}^2\right]$$

$$+ \sum_{j=0}^{\infty} \sum_{k \neq j+\tau_1} \sum_{l \neq j+\tau_1, l \neq k} 6\psi_j \psi_{j+\tau_2} \psi_k \psi_{k+\tau_2-\tau_1} \psi_l \psi_{l+\tau_2-\tau_1} \mathrm{E}\left[z_t^2 z_{t+j-k+\tau_1}^2 z_{t+j-l+\tau_1}^2\right]$$

$$\mathrm{E}\left[x_t x_{t+\tau_1}^2 x_{t+\tau_2}^2 x_{t+\tau_3}\right] = \sum_{j=0}^{\infty} \psi_j \psi_{j+\tau_1}^2 \psi_{j+\tau_2}^2 \psi_{j+\tau_3} \mathrm{E}\left[z_t^6\right] + \sum_{j=0}^{\infty} \sum_{k \neq j+\tau_2} \left(2\psi_j \psi_{j+\tau_1}^2\right.$$

$$\left.\psi_{j+\tau_2} \psi_k \psi_{k+\tau_3-\tau_2} + \psi_j \psi_{j+\tau_1}^2 \psi_{j+\tau_3} \psi_k^2\right) \mathrm{E}\left[z_t^4 z_{t+j-k+\tau_2}^2\right]$$

$$+ \sum_{j=0}^{\infty} \sum_{k \neq j+\tau_1} \left(\left(2\psi_j \psi_{j+\tau_1} \psi_{j+\tau_2}^2 \psi_k \psi_{k+\tau_3-\tau_1} + 4\psi_j \psi_{j+\tau_1} \psi_{j+\tau_2} \psi_{j+\tau_3} \psi_k \psi_{k+\tau_2-\tau_1}\right.\right.$$

$$\left.+\psi_j \psi_{j+\tau_2}^2 \psi_{j+\tau_3} \psi_k^2\right) \mathrm{E}\left[z_t^4 z_{t+j-k+\tau_1}^2\right] + \left(2\psi_j \psi_{j+\tau_1} \psi_k \psi_{k+\tau_2-\tau_1}^2 \psi_{k+\tau_3-\tau_1}\right.$$

$$\left.\left.+2\psi_j \psi_{j+\tau_2} \psi_k^2 \psi_{k+\tau_2-\tau_1} \psi_{k+\tau_3-\tau_1} + \psi_j \psi_{j+\tau_3} \psi_k^2 \psi_{k+\tau_2-\tau_1}^2\right) \mathrm{E}\left[z_t^2 z_{t+j-k+\tau_1}^4\right]\right)$$

$$+ \sum_{j=0}^{\infty} \sum_{k \neq j+\tau_1} \sum_{l \neq j+\tau_2, l \neq k+\tau_2-\tau_1} (4\psi_j \psi_{j+\tau_1} \psi_k \psi_{k+\tau_2-\tau_1} \psi_l \psi_{l+\tau_3-\tau_2} + 2\psi_j \psi_{j+\tau_1}$$

$$\left.\times \psi_k \psi_{k+\tau_3-\tau_1} \psi_l^2 + 2\psi_j \psi_{j+\tau_2} \psi_k^2 \psi_l \psi_{l+\tau_3-\tau_2} + \psi_j \psi_{j+\tau_3} \psi_k^2 \psi_l^2\right)$$

$$\times \mathrm{E}\left[z_t^2 z_{t+j-k+\tau_1}^2 z_{t+j-l+\tau_2}^2\right] + \sum_{j=0}^{\infty} \sum_{k \neq j+\tau_1} \sum_{l \neq j+\tau_1, l \neq k} (4\psi_j \psi_{j+\tau_2} \psi_k \psi_{k+\tau_2-\tau_1}$$

$$\times \psi_l \psi_{l+\tau_3-\tau_1} + 2\psi_j \psi_{j+\tau_3} \psi_k \psi_{k+\tau_2-\tau_1} \psi_l \psi_{l+\tau_2-\tau_1}) \mathrm{E}\left[z_t^2 z_{t+j-k+\tau_1}^2 z_{t+j-l+\tau_1}^2\right]$$

$$\mathrm{E}\left[x_t x_{t+\tau_1}^2 x_{t+\tau_2}\right] = \sum_{j=0}^{\infty} \psi_j \psi_{j+\tau_1}^2 \psi_{j+\tau_2} \mathrm{E}\left[z_t^4\right] + \sum_{j=0}^{\infty} \sum_{k \neq j+\tau_1} (2\psi_j \psi_{j+\tau_1} \times$$

$$\psi_k \psi_{k+\tau_2-\tau_1} + 2\psi_j \psi_{j+\tau_2} \psi_k^2\big) \mathrm{E}\left[z_t^2 z_{t+j-k+\tau_1}^2\right]$$

$$\mathrm{E}\left[x_t x_{t+\tau_1}^2 x_{t+\tau_2} x_{t+\tau_3}^2\right] = \sum_{j=0}^{\infty} \psi_j \psi_{j+\tau_1}^2 \psi_{j+\tau_2} \psi_{j+\tau_3}^2 \mathrm{E}\left[z_t^6\right] + \sum_{j=0}^{\infty} \sum_{k \neq j+\tau_3} \psi_j \psi_{j+\tau_1}^2 \psi_{j+\tau_2}$$

$$\times \psi_k^2 \mathrm{E}\left[z_t^4 z_{t+j-k+\tau_3}^2\right] + \sum_{j=0}^{\infty} \sum_{k \neq j+\tau_2} 2\psi_j \psi_{j+\tau_1}^2 \psi_{j+\tau_3} \psi_k \psi_{k+\tau_3-\tau_2} \mathrm{E}\left[z_t^4 z_{t+j-k+\tau_2}^2\right]$$

$$+ \sum_{j=0}^{\infty} \sum_{k \neq j+\tau_1} \big(\big(4\psi_j \psi_{j+\tau_1} \psi_{j+\tau_2} \psi_{j+\tau_3} \psi_k \psi_{k+\tau_3-\tau_1} + 2\psi_j \psi_{j+\tau_1} \psi_{j+\tau_3}^2 \psi_k \psi_{k+\tau_2-\tau_1}$$

$$+ \psi_j \psi_{j+\tau_2} \psi_{j+\tau_3}^2 \psi_k^2\big) \mathrm{E}\left[z_t^4 z_{t+j-k+\tau_1}^2\right] + \big(2\psi_j \psi_{j+\tau_1} \psi_k \psi_{k+\tau_2-\tau_1} \psi_{k+\tau_3-\tau_1}^2$$

$$+ \psi_j \psi_{j+\tau_2} \psi_k^2 \psi_{k+\tau_3-\tau_1}^2 + 2\psi_j \psi_{j+\tau_3} \psi_k^2 \psi_{k+\tau_2-\tau_1} \psi_{k+\tau_3-\tau_1}\big) \mathrm{E}\left[z_t^2 z_{t+j-k+\tau_1}^4\right]\big)$$

$$+ \sum_{j=0}^{\infty} \sum_{k \neq j+\tau_1} \sum_{l \neq j+\tau_3, l \neq k+\tau_3-\tau_1} \big(2\psi_j \psi_{j+\tau_1} \psi_k \psi_{k+\tau_2-\tau_1} \psi_l^2 + \psi_j \psi_{j+\tau_2} \psi_k^2 \psi_l^2\big)$$

$$\times \mathrm{E}\left[z_t^2 z_{t+j-k+\tau_1}^2 z_{t+j-l+\tau_2}^2\right] + \sum_{j=0}^{\infty} \sum_{k \neq j+\tau_1} \sum_{l \neq j+\tau_2, l \neq k+\tau_2-\tau_1} (4\psi_j \psi_{j+\tau_1} \psi_k \psi_{k+\tau_3-\tau_1}$$

$$\times \psi_l \psi_{l+\tau_3-\tau_2} + 2\psi_j \psi_{j+\tau_3} \psi_k^2 \psi_l \psi_{l+\tau_3-\tau_2}\big) \mathrm{E}\left[z_t^2 z_{t+j-k+\tau_1}^2 z_{t+j-l+\tau_2}^2\right]$$

$$+ \sum_{j=0}^{\infty} \sum_{k \neq j+\tau_1} \sum_{l \neq j+\tau_1, l \neq k} (2\psi_j \psi_{j+\tau_2} \psi_k \psi_{k+\tau_3-\tau_1} \psi_l \psi_{l+\tau_3-\tau_1} + 4\psi_j \psi_{j+\tau_3} \psi_k \psi_{k+\tau_2-\tau_1}$$

$$\times \psi_l \psi_{l+\tau_3-\tau_1}) \mathrm{E}\left[z_t^2 z_{t+j-k+\tau_1}^2 z_{t+j-l+\tau_1}^2\right]$$

$$\mathrm{E}\left[x_t x_{t+\tau}\right] = \sum_{j=0}^{\infty} \psi_j \psi_{j+\tau}$$

$$\mathrm{E}\left[x_t x_{t+\tau_1} x_{t+\tau_2}^4\right] = \sum_{j=0}^{\infty} \psi_j \psi_{j+\tau_1} \psi_{j+\tau_2}^4 \mathrm{E}\left[z_t^6\right] + \sum_{j=0}^{\infty} \sum_{k \neq j+\tau_2} \left(6\psi_j \psi_{j+\tau_1} \psi_{j+\tau_2}^2 \psi_k^2\right.$$

$$\times \mathrm{E}\left[z_t^4 z_{t+j-k+\tau_2}^2\right] + \psi_j \psi_{j+\tau_1} \psi_k^4 \mathrm{E}\left[z_t^2 z_{t+j-k+\tau_2}^4\right]\Big) + \sum_{j=0}^{\infty} \sum_{k \neq j+\tau_1} \left(4\psi_j \psi_{j+\tau_2}^3 \psi_k\right.$$

$$\times \psi_{k+\tau_2-\tau_1} \mathrm{E}\left[z_t^4 z_{t+j-k+\tau_1}^2\right] + 4\psi_j \psi_{j+\tau_2} \psi_k \psi_{k+\tau_2-\tau_1}^3 \mathrm{E}\left[z_t^2 z_{t+j-k+\tau_1}^4\right]\Big)$$

$$+ \sum_{j=0}^{\infty} \sum_{k \neq j+\tau_2} \sum_{l \neq j+\tau_2, l \neq k} 3\psi_j \psi_{j+\tau_1} \psi_k^2 \psi_l^2 \mathrm{E}\left[z_t^2 z_{t+j-k+\tau_2}^2 z_{t+j-l+\tau_2}^2\right]$$

$$+ \sum_{j=0}^{\infty} \sum_{k \neq j+\tau_1} \sum_{l \neq j+\tau_2, l \neq k+\tau_2-\tau_1} 12\psi_j \psi_{j+\tau_2} \psi_k \psi_{k+\tau_2-\tau_1} \psi_l^2 \mathrm{E}\left[z_t^2 z_{t+j-k+\tau_1}^2 z_{t+j-l+\tau_2}^2\right]$$

$$\mathrm{E}\left[x_t x_{t+\tau_1} x_{t+\tau_2}^2\right] = \sum_{j=0}^{\infty} \psi_j \psi_{j+\tau_1} \psi_{j+\tau_2}^2 \mathrm{E}\left[z_t^4\right] + \sum_{j=0}^{\infty} \sum_{k \neq j+\tau_2} \psi_j \psi_{j+\tau_1} \psi_k^2$$

$$\times \mathrm{E}\left[z_t^2 z_{t+j-k+\tau_2}^2\right] + \sum_{j=0}^{\infty} \sum_{k \neq j+\tau_1} 2\psi_j \psi_{j+\tau_2} \psi_k \psi_{k+\tau_2-\tau_1} \mathrm{E}\left[z_t^2 z_{t+j-k+\tau_1}^2\right]$$

$$
\begin{aligned}
&\mathrm{E}\left[x_t x_{t+\tau_1} x_{t+\tau_2}^2 x_{t+\tau_3}^2\right] = \sum_{j=0}^{\infty} \psi_j \psi_{j+\tau_1} \psi_{j+\tau_2}^2 \psi_{j+\tau_3}^2 \mathrm{E}\left[z_t^6\right] + \sum_{j=0}^{\infty} \sum_{k \neq j+\tau_3} \psi_j \psi_{j+\tau_1} \\
&\times \psi_{j+\tau_2}^2 \psi_k^2 \mathrm{E}\left[z_t^4 z_{t+j-k+\tau_3}^2\right] + \sum_{j=0}^{\infty} \sum_{k \neq j+\tau_2} ((4\psi_j \psi_{j+\tau_1} \psi_{j+\tau_2} \psi_{j+\tau_3} \psi_k \psi_{k+\tau_3-\tau_2} \\
&+\psi_j \psi_{j+\tau_1} \psi_{j+\tau_3}^2 \psi_k^2\big) \mathrm{E}\left[z_t^4 z_{t+j-k+\tau_2}^2\right] + \psi_j \psi_{j+\tau_1} \psi_k^2 \psi_{k+\tau_3-\tau_2}^2 \mathrm{E}\left[z_t^2 z_{t+j-k+\tau_2}^4\right]\big) \\
&+ \sum_{j=0}^{\infty} \sum_{k \neq j+\tau_1} \big(\big(2\psi_j \psi_{j+\tau_2}^2 \psi_{j+\tau_3} \psi_k \psi_{k+\tau_3-\tau_1} + 2\psi_j \psi_{j+\tau_2} \psi_{j+\tau_3}^2 \psi_k \psi_{k+\tau_2-\tau_1}\big) \\
&\times \mathrm{E}\left[z_t^4 z_{t+j-k+\tau_1}^2\right] + \big(2\psi_j \psi_{j+\tau_2} \psi_k \psi_{k+\tau_2-\tau_1} \psi_{k+\tau_3-\tau_1}^2 + 2\psi_j \psi_{j+\tau_3} \psi_k \psi_{k+\tau_2-\tau_1}^2 \\
&\times \psi_{k+\tau_3-\tau_1}\big) \mathrm{E}\left[z_t^2 z_{t+j-k+\tau_1}^4\right]\big) + \sum_{j=0}^{\infty} \sum_{k \neq j+\tau_2} \sum_{l \neq j+\tau_3, l \neq k+\tau_3-\tau_2} \psi_j \psi_{j+\tau_1} \psi_k^2 \psi_l^2 \\
&\times \mathrm{E}\left[z_t^2 z_{t+j-k+\tau_2}^2 z_{t+j-l+\tau_3}^2\right] + \sum_{j=0}^{\infty} \sum_{k \neq j+\tau_1} \sum_{l \neq j+\tau_3, l \neq k+\tau_3-\tau_1} 2\psi_j \psi_{j+\tau_2} \psi_k \psi_{k+\tau_2-\tau_1} \\
&\times \psi_l^2 \mathrm{E}\left[z_t^2 z_{t+j-k+\tau_1}^2 z_{t+j-l+\tau_3}^2\right] + \sum_{j=0}^{\infty} \sum_{k \neq j+\tau_2} \sum_{l \neq j+\tau_2, l \neq k} 2\psi_j \psi_{j+\tau_1} \psi_k \psi_{k+\tau_3-\tau_2} \psi_l \\
&\times \psi_{l+\tau_3-\tau_2} \mathrm{E}\left[z_t^2 z_{t+j-k+\tau_2}^2 z_{t+j-l+\tau_2}^2\right] + \sum_{j=0}^{\infty} \sum_{k \neq j+\tau_1} \sum_{l \neq j+\tau_2, l \neq k+\tau_2-\tau_1} (4\psi_j \psi_{j+\tau_2} \\
&\times \psi_k \psi_{k+\tau_3-\tau_1} \psi_l \psi_{l+\tau_3-\tau_2} + 4\psi_j \psi_{j+\tau_3} \psi_k \psi_{k+\tau_2-\tau_1} \psi_l \psi_{l+\tau_3-\tau_2} + 2\psi_j \psi_{j+\tau_3} \psi_k \\
&\times \psi_{k+\tau_3-\tau_1} \psi_l^2\big) \mathrm{E}\left[z_t^2 z_{t+j-k+\tau_1}^2 z_{t+j-l+\tau_2}^2\right]
\end{aligned}
$$

Proof of Theorem 4

(i): Write $\boldsymbol{V} = (\boldsymbol{v}_1, \ldots, \boldsymbol{v}_p)^T$ and $\boldsymbol{v}_i = (v_{i1}, \ldots, v_{ip})^T$. By using the basic properties of the covariance, we have that

$$\begin{aligned}
\boldsymbol{G}_m(\boldsymbol{V}\boldsymbol{x}) =& \sum_{i=1}^{p}\sum_{j=1}^{p} \mathrm{cov}^2(\boldsymbol{V}\boldsymbol{x}_t\boldsymbol{x}_t^T\boldsymbol{V}^T, (\boldsymbol{V}\boldsymbol{x})_{t-l,i}(\boldsymbol{V}\boldsymbol{x})_{t-l,j}) \\
=& \sum_{i=1}^{p}\sum_{j=1}^{p} \mathrm{cov}^2(\boldsymbol{V}\boldsymbol{x}_t\boldsymbol{x}_t^T\boldsymbol{V}^T, \boldsymbol{v}_i^T\boldsymbol{x}_{t-l}\boldsymbol{v}_j^T\boldsymbol{x}_{t-l}) \\
=& \sum_{i=1}^{p}\sum_{j=1}^{p} \mathrm{cov}(\boldsymbol{V}\boldsymbol{x}_t\boldsymbol{x}_t^T\boldsymbol{V}^T, \sum_{i'=1}^{p}(v_{ii'}x_{t-l,i'})\sum_{j'=1}^{p}(v_{jj'}x_{t-l,j'})) \\
& \mathrm{cov}(\boldsymbol{V}\boldsymbol{x}_t\boldsymbol{x}_t^T\boldsymbol{V}^T, \sum_{i''=1}^{p}(v_{ii''}x_{t-l,i''})\sum_{j''=1}^{p}(v_{jj''}x_{t-l,j''}))^T \\
=& \sum_{i=1}^{p}\sum_{j=1}^{p}\sum_{i'=1}^{p}\sum_{j'=1}^{p}\sum_{i''=1}^{p}\sum_{j''=1}^{p} \mathrm{cov}(\boldsymbol{V}\boldsymbol{x}_t\boldsymbol{x}_t^T\boldsymbol{V}^T, v_{ii'}v_{jj'}x_{t-l,i'}x_{t-l,j'}) \\
& \mathrm{cov}(\boldsymbol{V}\boldsymbol{x}_t\boldsymbol{x}_t^T\boldsymbol{V}^T, v_{ii''}v_{jj''}x_{t-l,i''}x_{t-l,j''})^T \\
=& \sum_{i=1}^{p}\sum_{j=1}^{p}\sum_{i'=1}^{p}\sum_{j'=1}^{p}\sum_{i''=1}^{p}\sum_{j''=1}^{p} v_{ii'}v_{jj'}v_{ii''}v_{jj''}\mathrm{cov}(\boldsymbol{V}\boldsymbol{x}_t\boldsymbol{x}_t^T\boldsymbol{V}^T, x_{t-l,i'}x_{t-l,j'}) \\
& \mathrm{cov}(\boldsymbol{V}\boldsymbol{x}_t\boldsymbol{x}_t^T\boldsymbol{V}^T, x_{t-l,i''}x_{t-l,j''})^T \\
=& \sum_{i'=1}^{p}\sum_{j'=1}^{p}\sum_{i''=1}^{p}\sum_{j''=1}^{p} \mathrm{cov}(\boldsymbol{V}\boldsymbol{x}_t\boldsymbol{x}_t^T\boldsymbol{V}^T, x_{t-l,i'}x_{t-l,j'})\mathrm{cov}(\boldsymbol{V}\boldsymbol{x}_t\boldsymbol{x}_t^T\boldsymbol{V}^T, x_{t-l,i''}x_{t-l,j''})^T \\
& \sum_{i=1}^{p}(v_{ii'}v_{ii''})\sum_{j=1}^{p}(v_{jj'}v_{jj''}).
\end{aligned}$$

Since $\boldsymbol{V}$ is orthogonal, $\sum_{i=1}^{p}(v_{ii'}v_{ii''}) = 1$, if $i' = i''$, and 0 otherwise. Hence,

$$\begin{aligned}
\boldsymbol{G}_m(\boldsymbol{V}\boldsymbol{x}) =& \sum_{i'=1}^{p}\sum_{j'=1}^{p} \mathrm{cov}(\boldsymbol{V}\boldsymbol{x}_t\boldsymbol{x}_t^T\boldsymbol{V}^T, x_{t-l,i'}x_{t-l,j'})\mathrm{cov}(\boldsymbol{V}\boldsymbol{x}_t\boldsymbol{x}_t^T\boldsymbol{V}^T, x_{t-l,i'}x_{t-l,j'}) \\
=& \sum_{i'=1}^{p}\sum_{j'=1}^{p} \boldsymbol{V}\mathrm{cov}(\boldsymbol{x}_t\boldsymbol{x}_t^T, x_{t-l,i'}x_{t-l,j'})\boldsymbol{V}^T\boldsymbol{V}\mathrm{cov}(\boldsymbol{x}_t\boldsymbol{x}_t^T, x_{t-l,i'}x_{t-l,j'})^T\boldsymbol{V}^T \\
=& \boldsymbol{V}\left(\sum_{i'=1}^{p}\sum_{j'=1}^{p} \mathrm{cov}^2(\boldsymbol{x}_t\boldsymbol{x}_t^T, x_{t-l,i'}x_{t-l,j'})\right)\boldsymbol{V}^T \\
=& \boldsymbol{V}\boldsymbol{G}_m(\boldsymbol{x})\boldsymbol{V}^T.
\end{aligned}$$

(ii): Let us write

$$\boldsymbol{G}_m(\boldsymbol{x}) = \sum_{l=1}^{m}\sum_{i=1}^{p}\sum_{j=1}^{p} \boldsymbol{g}_l^{ij}(\boldsymbol{x})\boldsymbol{g}_l^{ij}(\boldsymbol{x})^T,$$

where $\boldsymbol{g}_l^{ij}(\boldsymbol{x}) = \mathrm{cov}(\boldsymbol{x}_t\boldsymbol{x}_t^T, x_{t-l,i}x_{t-l,j})$. It is easy to see that

$$\boldsymbol{g}_l^{ii}(\boldsymbol{s})_{jk} = \mathrm{cov}(\boldsymbol{s}_{tj}\boldsymbol{s}_{tk}^T, s_{t-l,i}s_{t-l,i}) = 0,$$

if $j \neq k$, and thus $\boldsymbol{g}_l^{ii}(\boldsymbol{s})$ and $\boldsymbol{g}_l^{ii}(\boldsymbol{s})\boldsymbol{g}_l^{ii}(\boldsymbol{s})^T$ are diagonal. For $i \neq r$,

$$\boldsymbol{g}_l^{ir}(\boldsymbol{s})_{jk} = \mathrm{cov}(\boldsymbol{s}_{tj}\boldsymbol{s}_{tk}^T, s_{t-l,i}s_{t-l,r})$$

can be nonzero only if $j = i$ and $k = r$, or $j = r$ and $k = i$. This implies that the only possibly nonzero elements of $\boldsymbol{g}_l^{ir}(\boldsymbol{s})\boldsymbol{g}_l^{ir}(\boldsymbol{s})^T$ are the ith and rth diagonal elements. Therefore also $\boldsymbol{G}_m(\boldsymbol{s})$ is diagonal as a sum of diagonal matrices.
